# Supplementary material for: Chemotherapy-induced adipo-lineage cell senescence drives bone loss
Source: Nat Commun. 2025 Dec 30;17:1042. doi: 10.1038/s41467-025-67793-3 (PMC12848019; doi:10.1038/s41467-025-67793-3)
Supplement: Supplementary file 1 — Supplementary Information [file 41467_2025_67793_MOESM1_ESM.pdf]

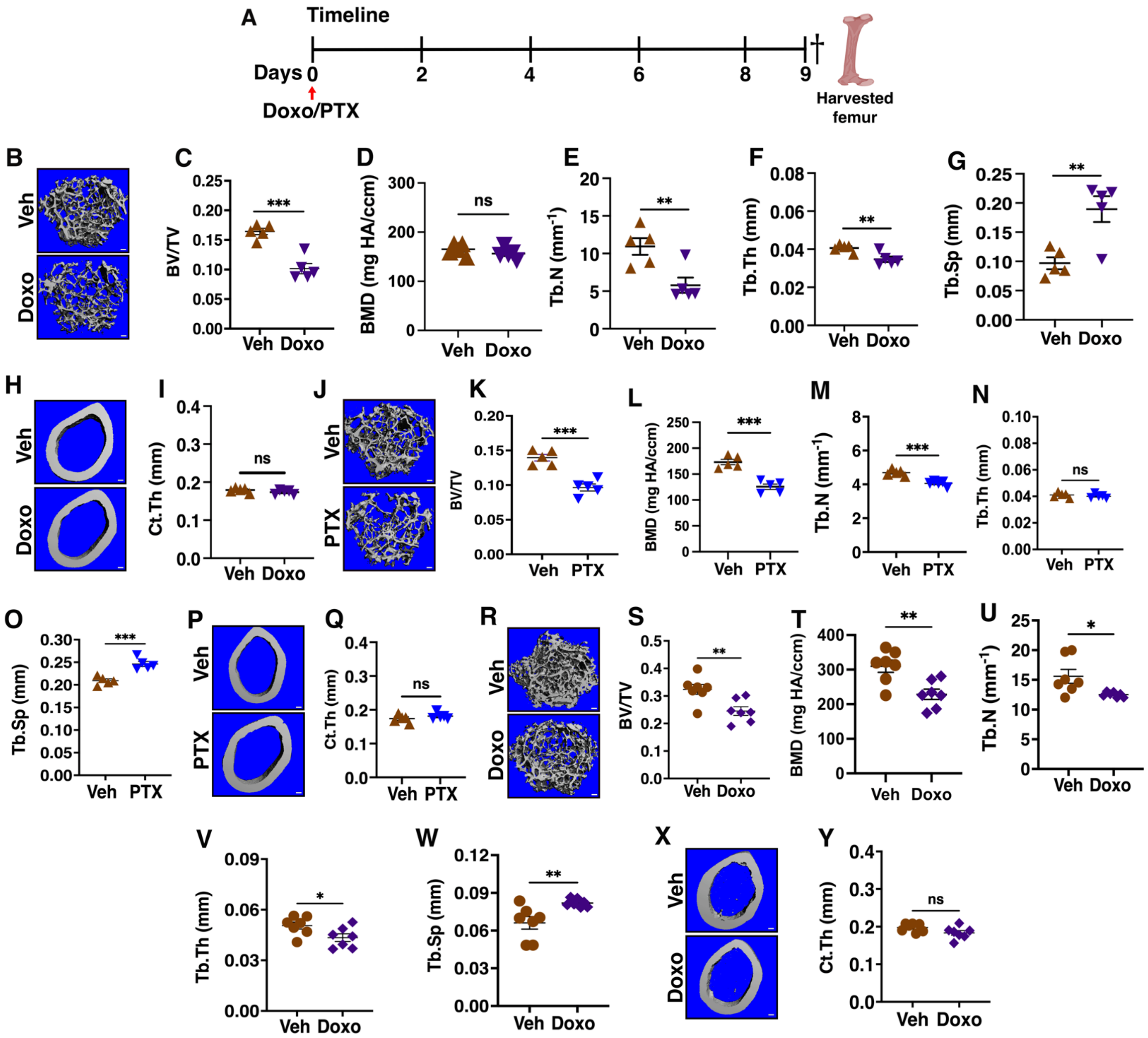

A
Timeline
Days 0 2 4 6 8 9
Doxo/PTX
Harvested femur
B C D E F G
Veh Doxo
BV/TV
BMD (mg HA/ccm)
Tb.N (mm-1)
Tb.Th (mm)
Tb.Sp (mm)
ns
H I J K L M N
Ct.Th (mm)
Veh PTX
O P Q R S T U
V W X Y

Fig. S1. Chemotherapy induces senescence and impacts bone homeostasis.

(A) Schematic showing experimental timeline for dosing regimen for doxorubicin (Doxo) or paclitaxel (PTX) in 12-week-old C57BL/6 wildtype mice. Dagger indicates time of sacrifice and bone harvest. Schematic was created in BioRender. Stewart, S. (2025) https://BioRender.com/71wz45j.

(B-Q) Representative μCT images of femurs and quantitative analyses of trabecular bone volume over total volume (BV/TV), bone mineral density (BMD), trabecular number (Tb. N), trabecular thickness (Tb. Th), trabecular spacing (Tb. Sp) and representative μCT images of cortical bone and quantitative analyses of cortical thickness (Ct. Th.) in 12-week-old female mice treated with Doxo (**B-I**) or PTX (**J-Q**). Scale bars:100μm. n = 5 mice/group.

(R-Y) Representative μCT images of femurs and quantitative analyses of trabecular bone volume over total volume (BV/TV), bone mineral density (BMD), trabecular number (Tb. N), trabecular thickness (Tb. Th), and trabecular separation (Tb.Sp) and representative μCT images of cortical bone and quantitative analyses of cortical thickness (Ct. Th.) in 12-week-old male mice treated with Veh or Doxo. Scale bars:100μm. n = 7 mice/group.

Data are represented as mean ± SEM. **P* < 0.05; ***P* < 0.01, ****P* < 0.001; *****P* < 0.0001; as determined by unpaired two-tailed Student’s *t*-test.

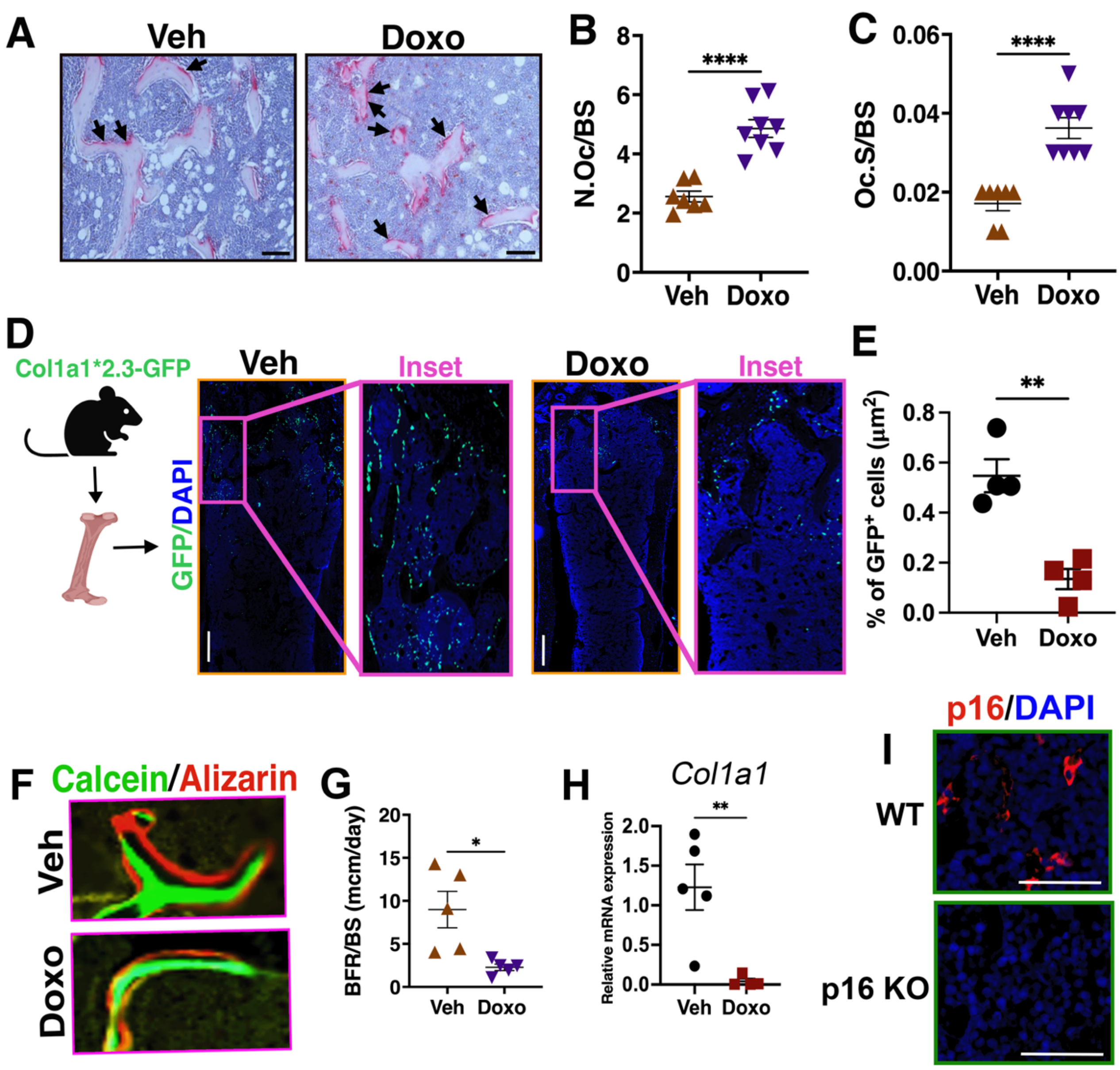

A
Veh
Doxo
B
N.Oc/BS
****
Veh
Doxo
C
Oc.S/BS
****
Veh
Doxo
D
Col1a1*2.3-GFP
GFP/DAPI
Veh
Inset
Doxo
Inset
E
% of GFP+ cells (μm2)
**
Veh
Doxo
F
Calcein/Alizarin
Veh
Doxo
G
BFR/BS (mcm/day)
*
Veh
Doxo
H
Col1a1
Relative mRNA expression
**
Veh
Doxo
I
p16/DAPI
WT
p16 KO

Fig. S2. Chemotherapy induces senescence and impacts bone homeostasis.

(A-C) Representative images showing TRAP+ osteoclasts (arrows). Quantification of the number of osteoclasts per bone surface (N.Oc/BS) and osteoclast surface area per bone surface (Oc.S/BS). Scale bar: 100μm. n = 7 mice (Veh) and 8 mice (Doxo).

(D and E) Representative images from Veh and Doxo treated mice showing Col1a1*2.3kb-GFP positive (osteoblast) cells and quantification of osteoblast number per bone surface. Scale bars: 500μm. Insets show the magnified view. Dotted line indicates the trabecular bone. n = 4 mice/group. Schematic was created in BioRender. Stewart, S. (2025) https://BioRender.com/71wz45j.

(F and G) Representative images of calcein and alizarin red double bone labeling and graph shows bone formation rate per bone surface (BFR/BS) in femurs under indicated conditions. n = 5 mice/group.

(H) mRNA expression of *Col1a1* as quantified by RT-qPCR in bone-resident fraction obtained from 12-week-old mice. TBP and tubulin were used as housekeeping genes. n = 5 mice (Veh) and 4 mice (Doxo).

(I) Femur sections from wildtype (WT) versus p16 knock-out mice (P16-KO) were stained with p16 primary antibody demonstrates p16 antibody specificity. Scale bar=50μm. n = 3 mice/group.

Data are represented as mean ± SEM. *P < 0.05; **P < 0.01, ****P < 0.0001, as determined by unpaired two-tailed Student's t-test.

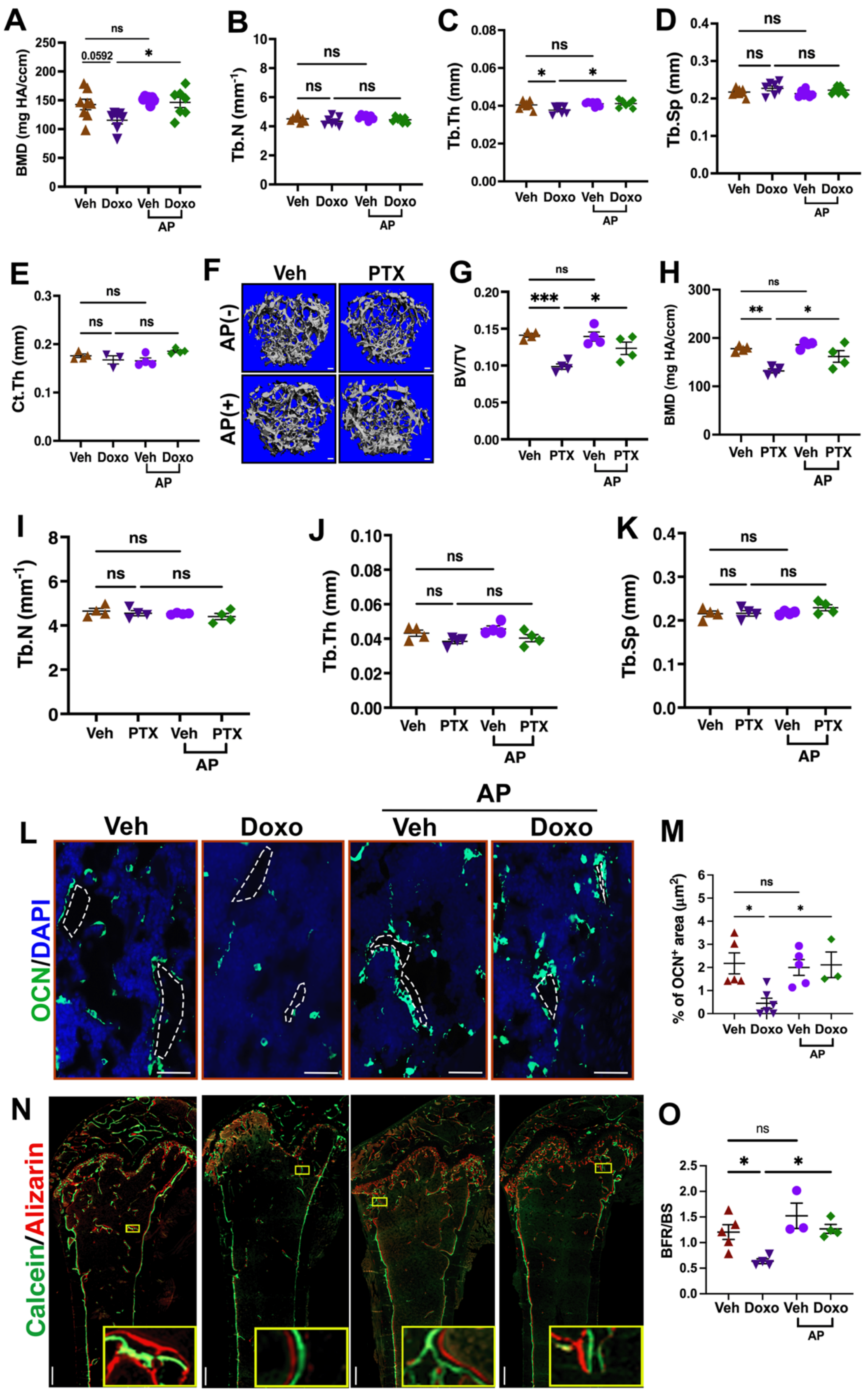
A
BMD (mg HA/ccm)
ns
0.0592
*
Veh Doxo Veh Doxo
AP
B
Tb.N (mm-1)
ns
ns ns
Veh Doxo Veh Doxo
AP
C
Tb.Th (mm)
ns
* *
Veh Doxo Veh Doxo
AP
D
Tb.Sp (mm)
ns
ns ns
Veh Doxo Veh Doxo
AP
E
Ct.Th (mm)
ns
ns ns
Veh Doxo Veh Doxo
AP
F
Veh PTX
AP(-)
AP(+)
G
BV/TV
ns
*** *
Veh PTX Veh PTX
AP
H
BMD (mg HA/ccm)
ns
** *
Veh PTX Veh PTX
AP
I
Tb.N (mm-1)
ns
ns ns
Veh PTX Veh PTX
AP
J
Tb.Th (mm)
ns
ns ns
Veh PTX Veh PTX
AP
K
Tb.Sp (mm)
ns
ns ns
Veh PTX Veh PTX
AP
AP
L
Veh Doxo Veh Doxo
OCN/DAPI
M
% of OCN+ area (µm2)
ns
* *
Veh Doxo Veh Doxo
AP
N
Calcein/Alizarin
O
BFR/BS
ns
* *
Veh Doxo Veh Doxo
AP

Fig. S3. Targeting senescent cells rescued chemotherapy-induced bone loss.

(A-E) 12-week-old INK-ATTAC female mice were administered a single dose of Doxo and subjected to bone mass measurements including bone mineral density (BMD), trabecular number (Tb. N), trabecular thickness (Tb. Th), trabecular spacing (Tb. Sp) and cortical thickness (Ct. Th.) with 3D reconstruction images using μCT.

(F-K) Representative μCT images of femurs and quantitative analyses of trabecular bone volume to total volume (BV/TV), BMD, trabecular number (Tb. N), trabecular thickness (Tb. Th), and trabecular separation (Tb.Sp) in 12-week-old female INK-ATTAC mice under paclitaxel (PTX) condition. Scale bars:100μm.

(L and M) IF staining of femur sections and quantification of the number of osteocalcin (OCN) positive area. Scale bar: 50μm. n = 5 mice (Veh), 6 (Doxo), 5 mice (Veh+AP), and 3 mice (Doxo+AP).

(N-O) Representative images of calcein and alizarin red double bone labeling and graph showing bone formation rate per bone surface (BFR/BS) in femurs. n = 5 mice (Veh), 4 (Doxo), 3 mice (Veh+AP), and 4 mice (Doxo+AP).

Data are represented as mean ± SEM. *P < 0.05; **P < 0.01; ***P < 0.001, ns = not significant as determined by unpaired two-tailed Student's t-test and one-way ANOVA with Tukey test.

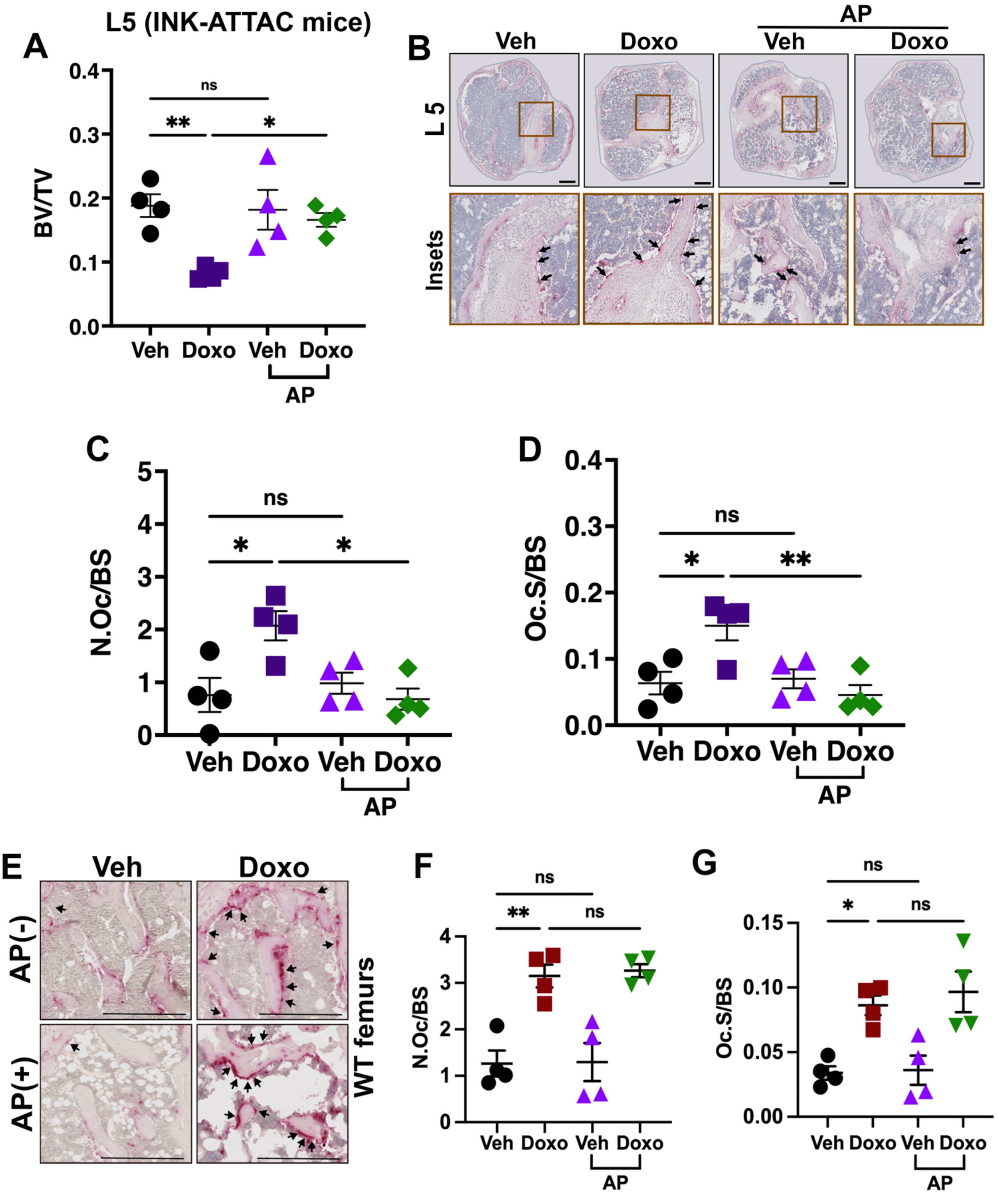
A
L5 (INK-ATTAC mice)
BV/TV
0.0
0.1
0.2
0.3
0.4
ns
**
*
Veh
Doxo
Veh
Doxo
AP
B
Veh
Doxo
AP
Veh
Doxo
L 5
Insets
C
N.Oc/BS
0
1
2
3
4
5
ns
*
*
Veh
Doxo
Veh
Doxo
AP
D
Oc.S/BS
0.0
0.1
0.2
0.3
0.4
ns
*
**
Veh
Doxo
Veh
Doxo
AP
E
Veh
Doxo
AP(-)
AP(+)
WT femurs
F
N.Oc/BS
0
1
2
3
4
ns
**
ns
Veh
Doxo
Veh
Doxo
AP
G
Oc.S/BS
0.00
0.05
0.10
0.15
ns
*
ns
Veh
Doxo
Veh
Doxo
AP

Fig. S4. Chemotherapy induces senescence in bone resident cells.

(A) μCT quantitative analyses of trabecular bone volume to total volume (BV/TV) for transplanted vertebral bodies (L5). n = 4 mice/group.

(B-D) TRAP staining of femur sections for osteoclasts (arrows) and quantification of the number of osteoclasts per bone surface (N.Oc/BS) and osteoclast surface area per bone surface (Oc.S/BS) for vertebral bodies (L5). n = 4 mice/group.

(E-G) TRAP staining using vertebral body implant-bearing recipient mice femurs. Representative images showing TRAP+ osteoclasts (arrows). Quantification of the number of osteoclasts per bone surface (N.Oc/BS) and osteoclast surface area per bone surface (Oc.S/BS). Scale bar: 100μm. n = 4 mice/group.

Data are represented as mean ± SEM. **P* < 0.05, ***P* < 0.01, ns = not significant as determined by unpaired two-tailed Student's *t*-test and one-way ANOVA with Tukey test.

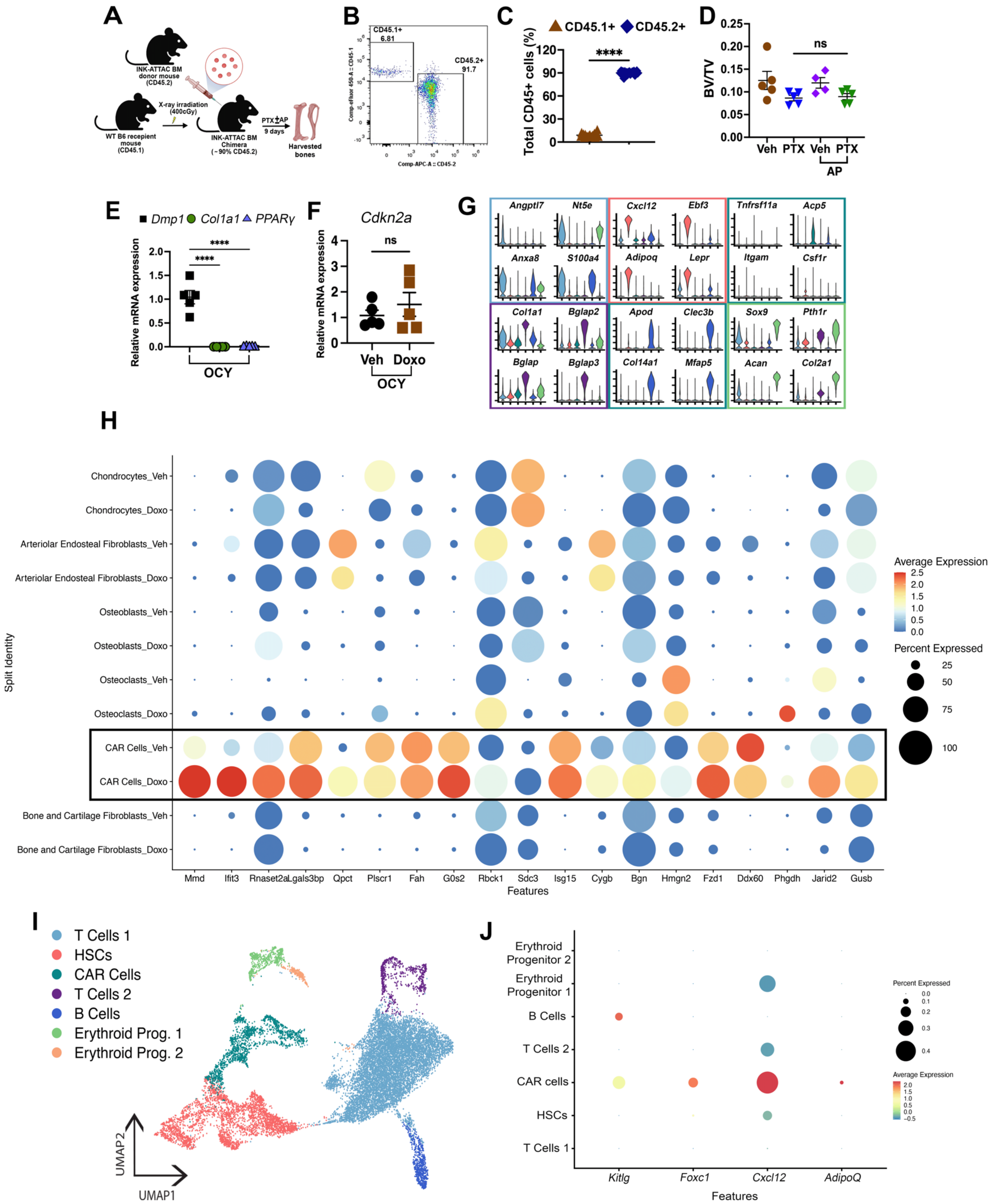
A
INK-ATTAC BM donor mouse (CD45.2)
X-ray irradiation (400cGy)
WT B6 recepient mouse (CD45.1)
INK-ATTAC BM Chimera (~90% CD45.2)
PTX±AP
9 days
Harvested bones
B
CD45.1+
6.81
CD45.2+
91.7
Comp-APC-A :: CD45-2
C
CD45.1+
CD45.2+
Total CD45+ cells (%)
****
D
BV/TV
ns
Veh
PTX
Veh
PTX
AP
E
Dmp1
Col1a1
PPARγ
Relative mRNA expression
****
****
OCY
F
Cdkn2a
Relative mRNA expression
ns
Veh
Doxo
OCY
G
Angptl7
Nt5e
Cxcl12
Ebf3
Tnfrsf11a
Acp5
Anxa8
S100a4
Adipoq
Lepr
Itgam
Csf1r
Col1a1
Bglap2
Apod
Clec3b
Sox9
Pth1r
Bglap
Bglap3
Col14a1
Mfap5
Acan
Col2a1
H
Chondrocytes_Veh
Chondrocytes_Doxo
Arteriolar Endosteal Fibroblasts_Veh
Arteriolar Endosteal Fibroblasts_Doxo
Osteoblasts_Veh
Osteoblasts_Doxo
Osteoclasts_Veh
Osteoclasts_Doxo
CAR Cells_Veh
CAR Cells_Doxo
Bone and Cartilage Fibroblast_Veh
Bone and Cartilage Fibroblasts_Doxo
Split Identity
Mmd
Ifit3
Rnaset2a
Lgals3bp
Qpct
Plscr1
Fah
G0s2
Rbck1
Sdc3
Isg15
Cygb
Bgn
Hmgn2
Fzd1
Ddx60
Phgdh
Jarid2
Gusb
Features
Average Expression
Percent Expressed
I
T Cells 1
HSCs
CAR Cells
T Cells 2
B Cells
Erythroid Prog. 1
Erythroid Prog. 2
UMAP1
UMAP2
J
Erythroid Progenitor 2
Erythroid Progenitor 1
B Cells
T Cells 2
CAR cells
HSCs
T Cells 1
Kitlg
Foxc1
Cxcl12
AdipoQ
Features
Percent Expressed
Average Expression

Fig. S5. Senescent bone resident cells drive chemotherapy-induced bone loss.

(A-C) Schematic illustrates bone marrow transplantation schema to generate mixed chimeras in mice (**A**) and shows the level of chimerism in recipient mice using flow cytometry (**B & C**). n = 10 mice/group. Schematic was created in BioRender. Stewart, S. (2025) https://BioRender.com/71wz45j.

(D) μCT analysis shows trabecular bone volume to total volume (BV/TV) in bone marrow transplanted model with indicated treatments. AP will only activate the suicide gene in p16$^+$ senescent CD45$^+$ cells if they exist. n = 5 mice (Veh), 4 mice (PTX), 4 mice (Veh+AP), and 5 mice (PTX+AP).

(E and F). Validation of osteocytes purification (**E**) and *Cdkn2a* gene expression in isolated osteocytes (**F**) by RT-qPCR from 12-weeks-old mice bone. Actin and cyclophilin were used as housekeeping genes. n = 5 mice/group.

(G) Violin density plots of cell markers defining cluster identities. Default assay was set to ALRA.

(H) Dot plot showing the CHICAS_RB1_TARGETS senescence signatures across the clusters. Default assay was set to ALRA.

(I) UMAP visualization of single cell transcriptomes displaying seven clusters in combined groups obtained from human bone samples.

(J) Dot plots of cell markers defining cluster identities including enriched CAR cell population in human bone samples.

Data are represented as mean ± SEM. ****$P < 0.0001$; ns = significant as determined by unpaired two-tailed Student's *t*-tests and one-way ANOVA with Tukey test.

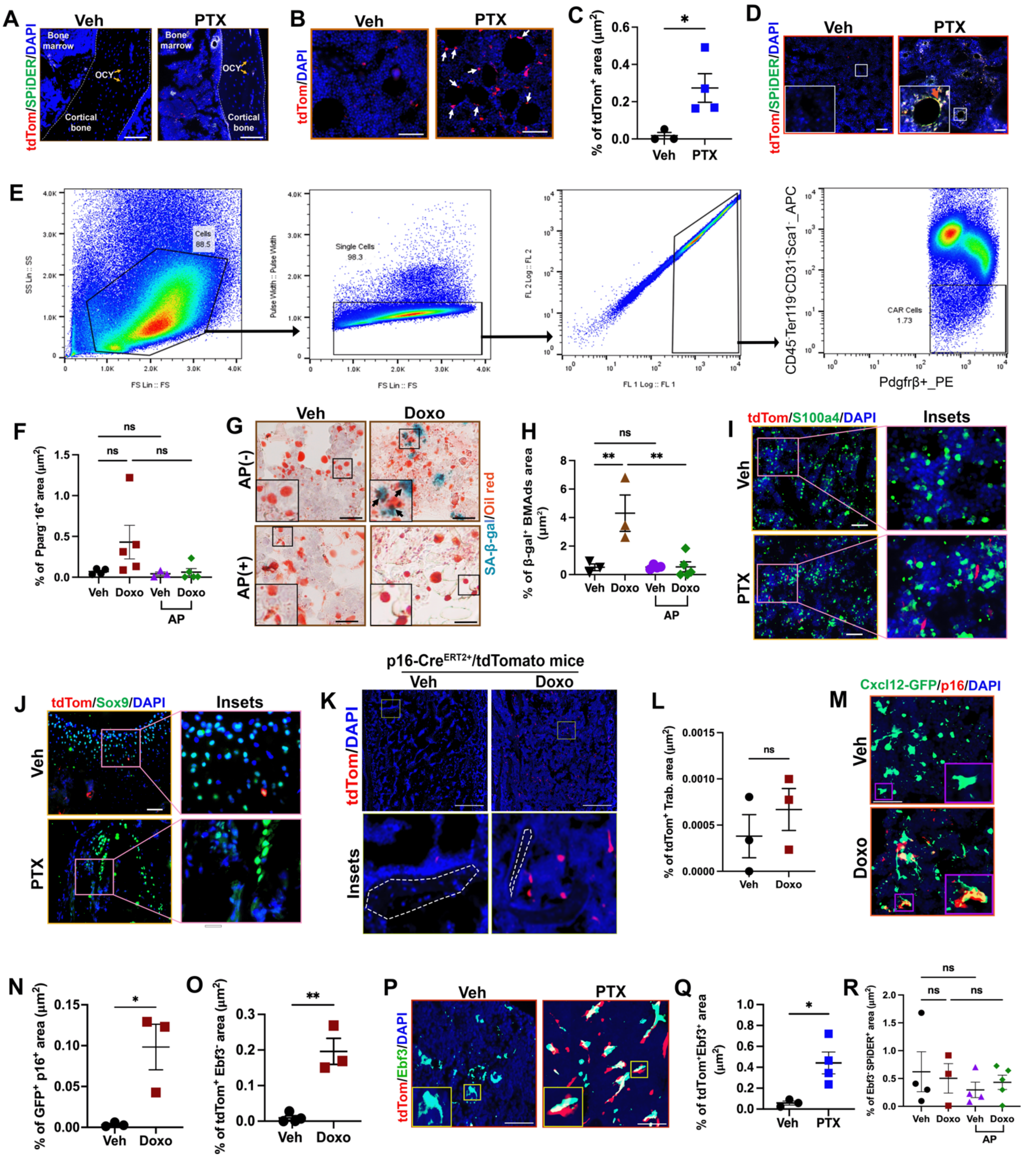
A
Veh
PTX
tdTom/SPiDER/DAPI
Bone marrow
OCY
Cortical bone
B
Veh
PTX
tdTom/DAPI
C
% of tdTom+ area (µm2)
Veh
PTX
D
Veh
PTX
tdTom/SPiDER/DAPI
E
Cells 88.5
Single Cells 98.3
CAR Cells 1.73
CD45-Ter119-CD31-Sca1-_APC
Pdgfrβ+_PE
F
% of Pparg+ 16+ area (µm2)
ns
Veh
Doxo
AP
G
Veh
Doxo
AP(-)
AP(+)
SA-β-gal/Oil red
H
% of β-gal+ BMAds area (µm2)
ns
**
Veh
Doxo
AP
I
tdTom/S100a4/DAPI
Insets
Veh
PTX
J
tdTom/Sox9/DAPI
Insets
Veh
PTX
K
p16-CreERT2+/tdTomato mice
Veh
Doxo
tdTom/DAPI
Insets
L
% of tdTom+ Trab. area (µm2)
ns
Veh
Doxo
M
Cxcl12-GFP/p16/DAPI
Veh
Doxo
N
% of GFP+ p16+ area (µm2)
*
Veh
Doxo
O
% of tdTom+ Ebf3+ area (µm2)
**
Veh
Doxo
P
Veh
PTX
tdTom/Ebf3/DAPI
Q
% of tdTom+Ebf3+ area (µm2)
*
Veh
PTX
R
% of Ebf3+SPiDER+ area (µm2)
ns
Veh
Doxo
AP

Fig. S6. Chemotherapy induces senescence in bone marrow adipo-lineage cells.

(A) Images show osteocytes (OCY) in cortical region after staining with SPiDER in *in vivo* lineage tracing p16-Cre^{ERT2}/tdTomato mice. Scale bar: 100μm. n = 4 mice/group.

(B and C) Representative images of tdTomato-expressing cells in distal femur of PTX treated 12-week-old p16-Cre^{ERT2}/tdTomato mice. DAPI stained nuclei are blue. Scale bar: 100μm. Arrows indicates tdTom signals (red). Quantification of the percentage tdTom+ area. n = 3 mice (Veh) and 4 mice (PTX).

(D) Representative IF staining for the detection of tdTom signal overlapped with SPiDER in adipocytes. Scale bar: 100μm. n = 3/group.

(E) Fluorescence-activated cell sorting (FACS) gating strategy for isolating CAR Cell-enriched population.

(F) Quantification of PPARg^{-} p16^{+} cells area in bone sections of p16-Cre^{ERT2}/tdTomato mice.

(G and H) Frozen femur sections from INK-ATTAC mice were subjected to SA-β-gal/oil red O co-staining in indicated groups. Representative images of SA-β-gal/oil red O positive cells (arrows) in metaphysis are shown in insets. Quantification of SA-β-gal positive bone marrow adipocyte (BMAd) area. Scale bar: 100μm. n = 3 mice (Veh), 3 (Doxo), 4 mice (Veh+AP), and 5 mice (Doxo+AP).

(I and J) Representative IF staining for the detection of colocalization of tdTom with S100a4 (**I**) and Sox9 (**J**) in indicated groups. Scale bar: 100μm. n = 3 mice/group.

(K and L) Representative IF staining for the detection of tdTom signal on the bone surface. Scale bar: 100μm and quantification (**L**). n = 3 mice/group.

(**M and N**) Representative IF staining for the detection of p16 colocalization with CAR cells in Cxcl12-GFP mice. Scale bar: 50μm. n = 3 mice/group.

(O) Quantification of $tdTom^{+}EBF3^{-}$ cell area in femurs.

(P and Q) Representative IF staining for the detection of tdTom signal overlapped with EBF3 and quantification of $tdTom^{+}EBF3^{+}$ area. Scale bar: 50μm. n = 3 mice (Veh) and 4 mice (PTX).

(R) Quantification of $EBF3^{-}$ $SPiDER^{+}$ cell area in femurs.

Data are represented as mean ± SEM. *P < 0.05; **P < 0.01; ns = not significant as determined by unpaired two-tailed Student's *t*-tests and one-way ANOVA with Tukey test.

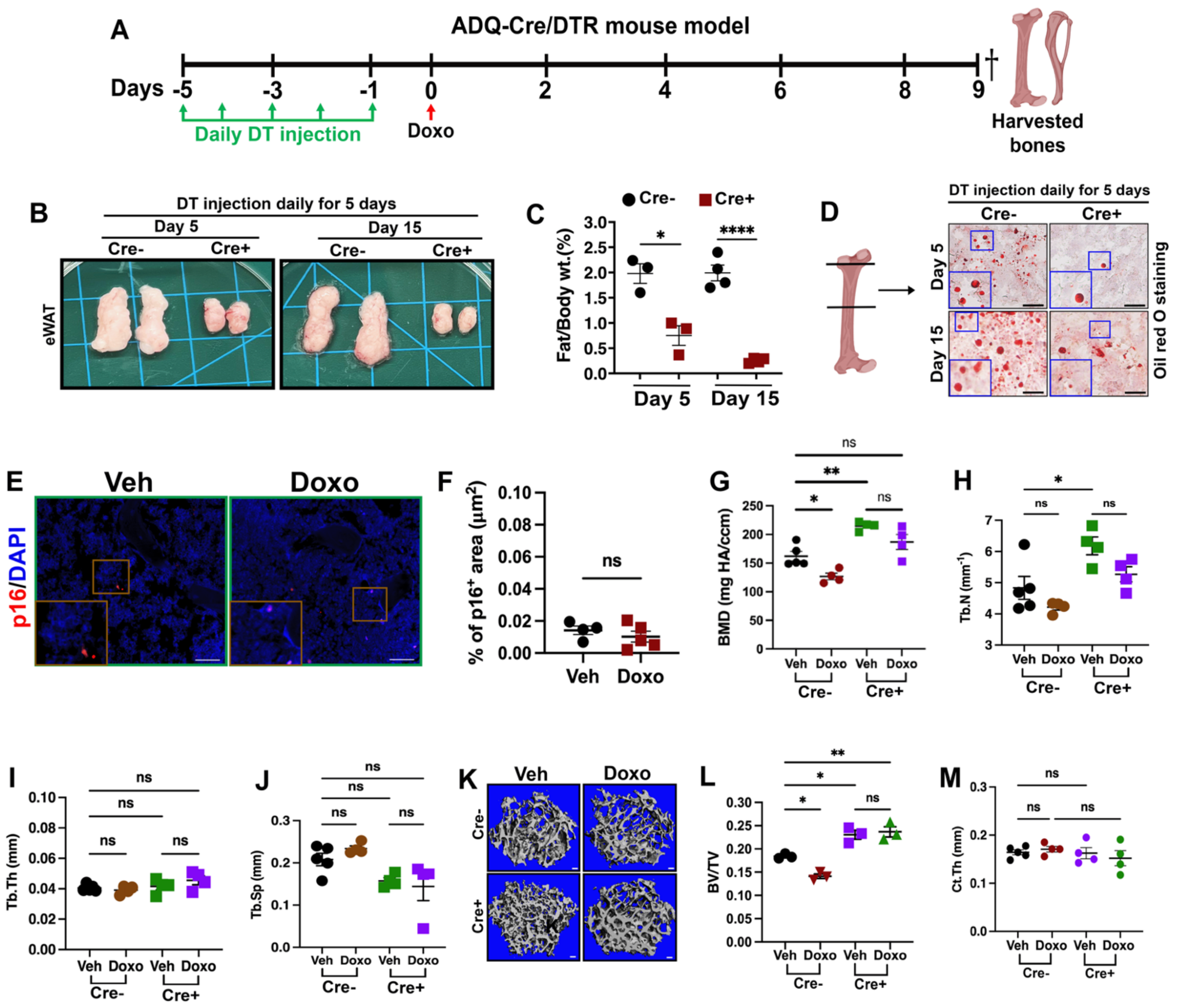

A
ADQ-Cre/DTR mouse model
Days -5 -3 -1 0 2 4 6 8 9
Daily DT injection
Doxo
Harvested bones
B
DT injection daily for 5 days
Day 5
Day 15
Cre- Cre+
eWAT
C
Cre- Cre+
Fat/Body wt.(%)
Day 5 Day 15
D
DT injection daily for 5 days
Cre- Cre+
Day 5
Day 15
Oil red O staining
E
Veh Doxo
p16/DAPI
F
% of p16+ area (μm2)
Veh Doxo
G
BMD (mg HA/ccm)
H
Tb.N (mm-1)
I
Tb.Th (mm)
J
Tb.Sp (mm)
K
Veh Doxo
Cre- Cre+
L
BV/TV
M
Ct.Th (mm)

Fig. S7. Ablation of fat cells prevent chemotherapy-induced bone loss.

(A) The schematic illustrates the time points for diphtheria toxin and other treatments in ADQ-Cre/DTR mice. Schematic was created in BioRender. Stewart, S. (2025) https://BioRender.com/71wz45j.

(B and C) ADQ-Cre-/DTR$^{f/f}$ and ADQ-Cre+/DTR$^{f/f}$ mice received DT daily for 5 consecutive days virtually eliminates eWAT without fat recovery at day 15. Quantitation of fat/body weight (%) on the indicated days. n = 3 mice (Day 5) and 4 mice (Day 15).

(D) Oil red staining of femurs showing bone marrow adipocyte loss is maintained through day 15 following DT treatment. Scale bar: 100μm. n = 3 mice/group. Schematic was created in BioRender. Stewart, S. (2025) https://BioRender.com/71wz45j.

(E and F) Immunofluorescence staining for p16 in ADQ-Cre+/DTR$^{f/f}$ mice. Only Doxo-treated mice received DT. Scale bar: 50μm. n = 4 mice (Veh) and 5 mice (DOXO).

(G-J) Additional bone structural parameters in ADQ-Cre-/DTR$^{f/f}$ and ADQ-Cre+/DTR$^{f/f}$ mice. n = 4 mice (Veh) and 5 mice (DOXO).

(K and L) μCT 3D images and analysis shows trabecular bone volume to total volume (BV/TV) in male ADQ-Cre-/DTR$^{f/f}$ and ADQ-Cre+/DTR$^{f/f}$ mice. All mice received DT. n = 3 mice/group.

(M) Quantitative analysis of cortical thickness (Ct. Th.) in female ADQ-Cre-/DTR$^{f/f}$ and ADQ-Cre+/DTR$^{f/f}$ mice under indicated treatment. n = 4 mice (Veh) and 5 mice (DOXO).

Data are represented as mean ± SEM. *P < 0.05; **P < 0.01; ****P < 0.0001; ns = not significant as determined by unpaired two-tailed Student's *t*-tests and one-way ANOVA with Tukey test.

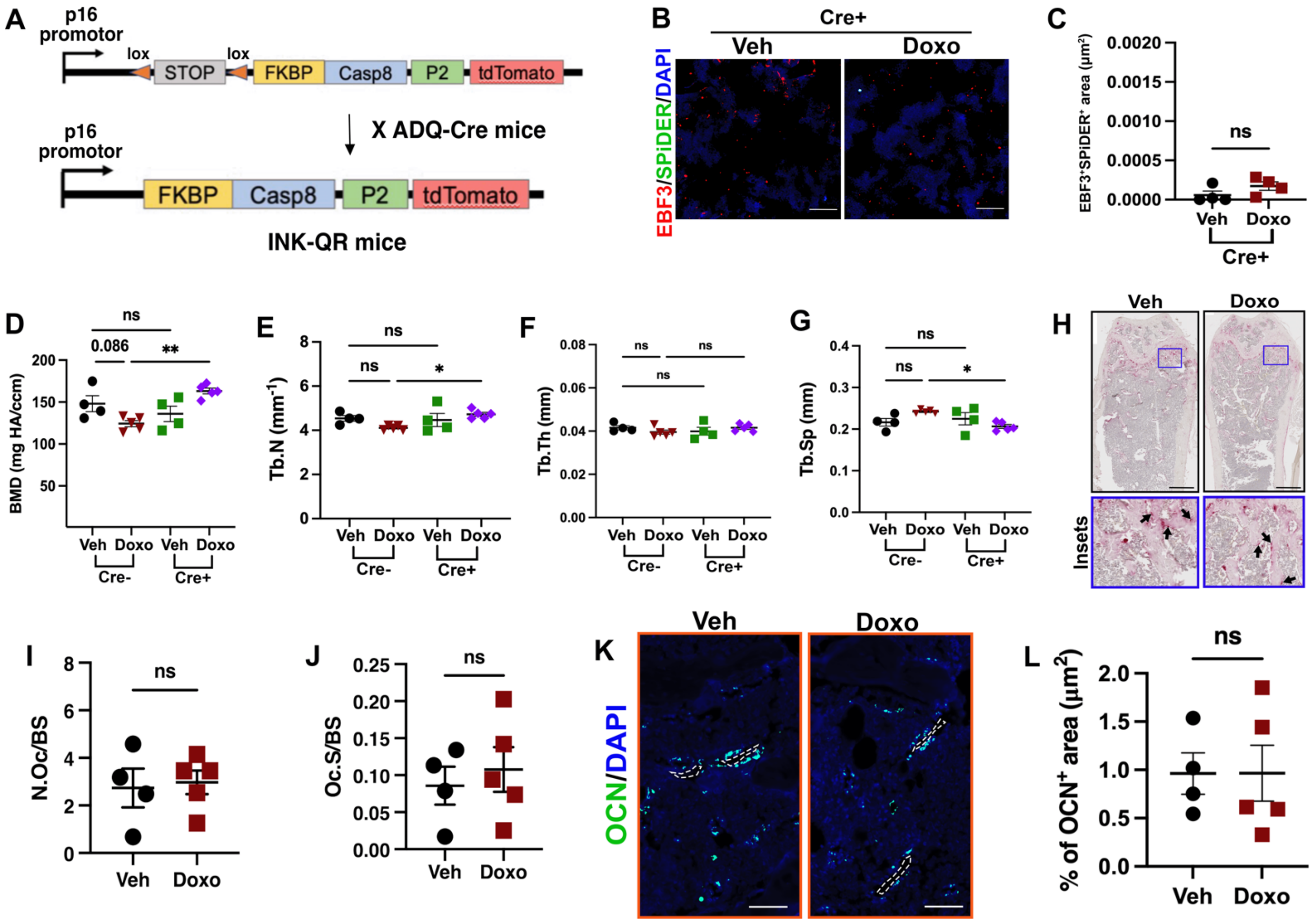

A
p16 promotor
lox
STOP
lox
FKBP
Casp8
P2
tdTomato
X ADQ-Cre mice
INK-QR mice
B
Cre+
Veh
Doxo
EBF3/SPiDER/DAPI
C
EBF3+SPiDER+ area (μm2)
ns
Cre+
D
BMD (mg HA/ccm)
0.086
**
Cre-
E
Tb.N (mm-1)
*
F
Tb.Th (mm)
G
Tb.Sp (mm)
H
Insets
I
N.Oc/BS
J
Oc.S/BS
K
OCN/DAPI
L
% of OCN+ area (μm2)

Fig. S8. Elimination of ADQ+ senescent cells prevents chemotherapy-induced bone loss.

(A) The schematic illustrates the ADQ-Cre mouse construct and the breeding strategy used to generate experimental cohorts.

(B and C) Immunofluorescence staining and quantification for EBF3 and SPiDER in ADQ-Cre/QR mice. Scale bar: 50μm. n = 4 mice/group.

(D-G) Additional bone structural parameters obtained from ADQ-Cre/QR mice μCT data. n = 4 mice/group.

(H-J) TRAP staining of femur sections and quantification of the number of osteoclasts (arrows) in ADQ-Cre+/DTR$^{f/f}$ mice. Only Doxo-treated mice received DT. Scale bar: 100μm. Insets represents a magnified view of the osteoclasts. Quantification of the number of osteoclasts per bone surface (N.Oc/BS) and osteoclast surface area per bone surface (Oc.S/BS). n = 4 mice (Veh) and 5 mice (Doxo).

(K and L) Representative osteocalcin (OCN) immunofluorescence in ADQ-Cre+/DTR$^{f/f}$ mice. Only Doxo-treated mice received DT. Scale bar: 50μm. n = 4 mice (Veh) and 5 mice (Doxo).

Data are represented as mean ± SEM. *$P < 0.05$; **$P < 0.01$; ns = not significant as determined by unpaired two-tailed Student's *t*-tests and one-way ANOVA with Tukey test.

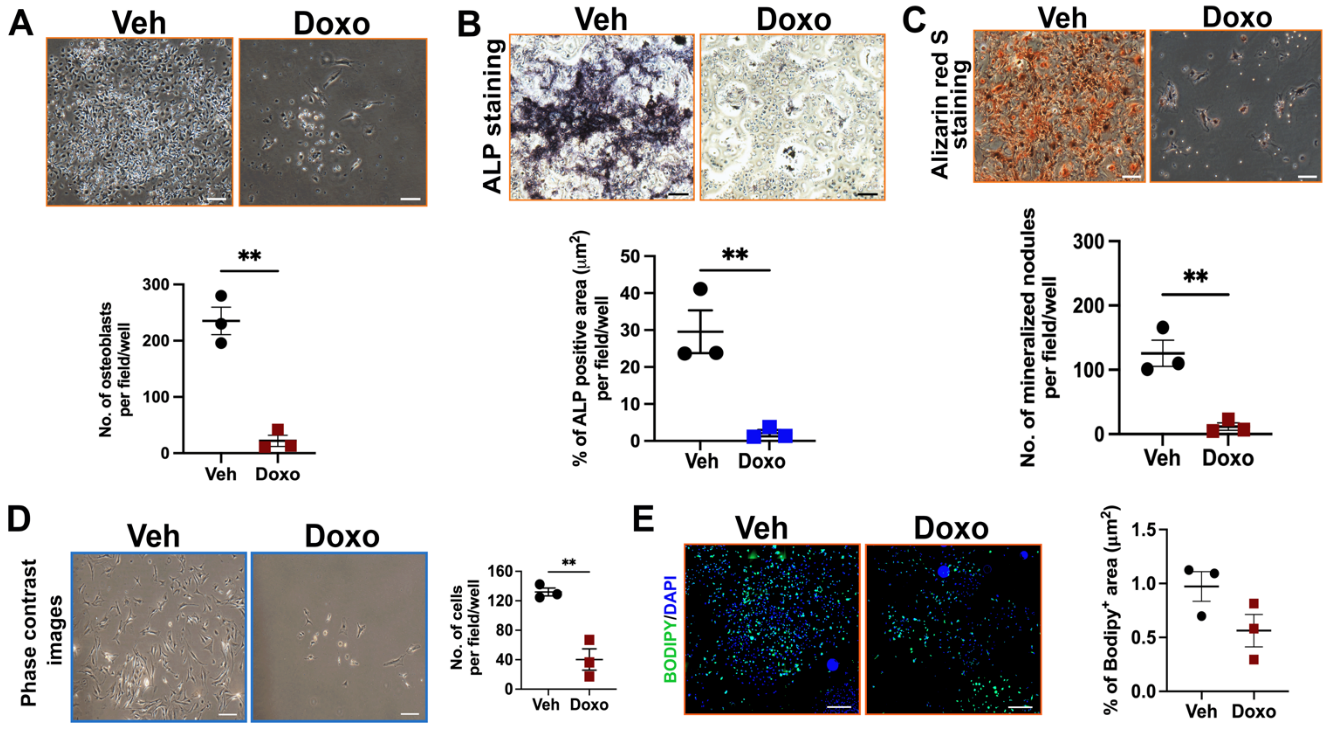

A
Veh
Doxo
No. of osteoblasts per field/well
300
200
100
0
**
Veh
Doxo
B
ALP staining
Veh
Doxo
% of ALP positive area (μm2) per field/well
50
40
30
20
10
0
**
Veh
Doxo
C
Alizarin red S staining
Veh
Doxo
No. of mineralized nodules per field/well
300
200
100
0
**
Veh
Doxo
D
Phase contrast images
Veh
Doxo
No. of cells per field/well
160
120
80
40
0
**
Veh
Doxo
E
BODIPY/DAPI
Veh
Doxo
% of Bodipy+ area (μm2)
1.5
1.0
0.5
0.0
Veh
Doxo

Fig. S9. Bone marrow derived from chemotherapy treated mice show reduced osteogenic potential.

(A) Bone marrow was isolated from Veh or Doxo treated mice at day nine and an *in vitro* osteoblast differentiation assay was performed. Phase contrast microscope images showing osteoblast differentiation at day 21. Images were taken with a 4x objective. Five fields were counted for each well and the graph represents the average number of osteoblasts per field. Experiment was performed in triplicate and each dot represents an independent sample. Scale bar: 100μm. Quantification of cells. n = 3 mice/group.

(B) Representative images of Alkaline phosphatase (ALP) staining at day 14. Graph represents % of ALP+ area per field. Images were taken with a 4x objective and 5 fields were counted for each well. Experiment was performed in triplicate and each dot represents an independent sample. Scale bar: 100μm. n = 3 mice/group.

(C) Alizarin red S staining for OB-mediated mineralization. The calcified nodules appeared bright red in color at day 21. Images were taken with a 4x objective and 5 fields were counted for each well. Graph represents average the number of mineralized nodules per field. Experiment was performed in triplicate and each dot represents an independent sample. Scale bar: 100μm. n = 3 mice/group.

(D) Phase-contrast images of mouse bone marrow cells isolated from Veh or Doxo treated mice and differentiated in the presence of adipogenic medium for 14 days. Scale bar: 50μm. Quantification of cells. n = 3 mice/group.

(E) Representative images of BODIPY staining of lipid droplets (green) during the bone marrow differentiation on culture day 14. Scale bar: 500μm. Quantification of percentage of BODIPY area. n = 3 mice/group.

Data are represented as mean ± SEM. $^{**}P < 0.01$ as determined by one-way ANOVA with Tukey test and unpaired two-tailed Student's *t*-tests.

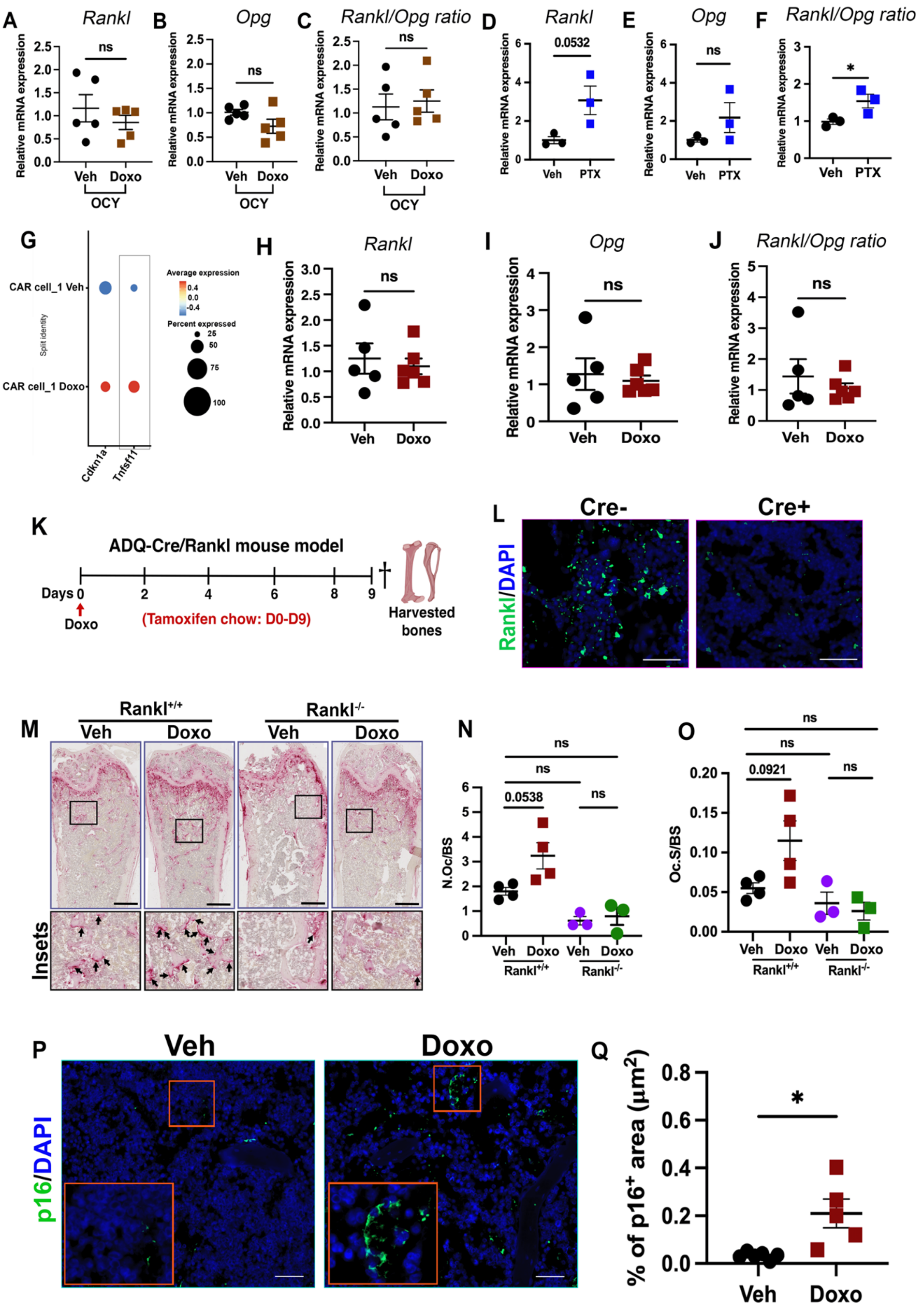
A
Rankl
B
Opg
C
Rankl/Opg ratio
D
Rankl
E
Opg
F
Rankl/Opg ratio
Relative mRNA expression
ns
0.0532
*
Veh
Doxo
OCY
PTX
G
CAR cell_1 Veh
CAR cell_1 Doxo
Split identity
Average expression
Percent expressed
Cdkn1a
Tnfsf11
H
Rankl
I
Opg
J
Rankl/Opg ratio
K
ADQ-Cre/Rankl mouse model
Days
Doxo
(Tamoxifen chow: D0-D9)
Harvested bones
L
Cre-
Cre+
Rankl/DAPI
M
Rankl+/+
Rankl-/-
Insets
N
N.Oc/BS
0.0538
O
Oc.S/BS
0.0921
P
p16/DAPI
Q
% of p16+ area (μm2)

Fig. S10. Senescent BM adipo-lineage cells drive bone loss through increase expression of RANKL.

(A-C) mRNA expression of *Rankl, Opg* and *Rankl/Opg ratio* as quantified by RT-qPCR in osteocyte enriched bone fractions from 12-week-old mice. Actin and cyclophilin were used as housekeeping genes. n = 5 mice/group.

(D-F) mRNA expression of *Rankl, Opg* and *Rankl/Opg ratio* as quantified by RT-qPCR using PTX-treated bone-resident fraction. TBP and tubulin were used as housekeeping genes. n = 3 mice/group.

(G) Dot plot demonstrating *Rankl* expression in Doxo-treated CAR cells versus Veh-treated CAR cells.

(H-J) mRNA expression of *Rankl, Opg and Rankl/Opg ratio* as quantified by RT-qPCR using bone-resident fraction of ADQ-Cre+/DTR mice. Only Doxo-treated mice received DT. Actin and cyclophilin were used as housekeeping genes. n = 5 mice/group.

(K) The schematic illustrates the time points for Doxo injection and other treatments in ADQ-Cre/Rankl mice. Schematic was created in BioRender. Stewart, S. (2025) https://BioRender.com/71wz45j.

(L) IF staining confirmed *Rankl* deletion in ADQ-Cre+/Rankl$^{fl/fl}$ mice femur. Green and blue colors represent RANKL and nucleus respectively. Scale bar: 50μm. n = 4 mice/group.

(M-O) TRAP staining of femurs from ADQ-Cre-/Rankl$^{fl/fl}$ and ADQ-Cre+/Rankl$^{f/f}$ mice. showing osteoclasts (arrows). Insets show magnified figures and arrows indicate pink colored osteoclasts. Quantification of the number of osteoclasts per bone surface

(N.Oc/BS) and osteoclast surface area per bone surface (Oc.S/BS). n = 4 mice ($Rankl^{+/+}$) and 3 mice ($Rankl^{-/-}$). Scale bar: 100µm.

(P and Q) Immunofluorescence staining for p16 in femur sections obtained from ADQ-Cre+/$Rankl^{fl/fl}$ mice. p16+ cells are visualized in green, and nuclei are counterstained with DAPI (blue). Scale bar: 50µm. n = 6 mice (Veh) and 5 mice (Doxo).

Data are represented as mean ± SEM. *$P < 0.05$; ns = not significant as determined by unpaired two-tailed Student's *t*-tests and one-way ANOVA with Tukey.

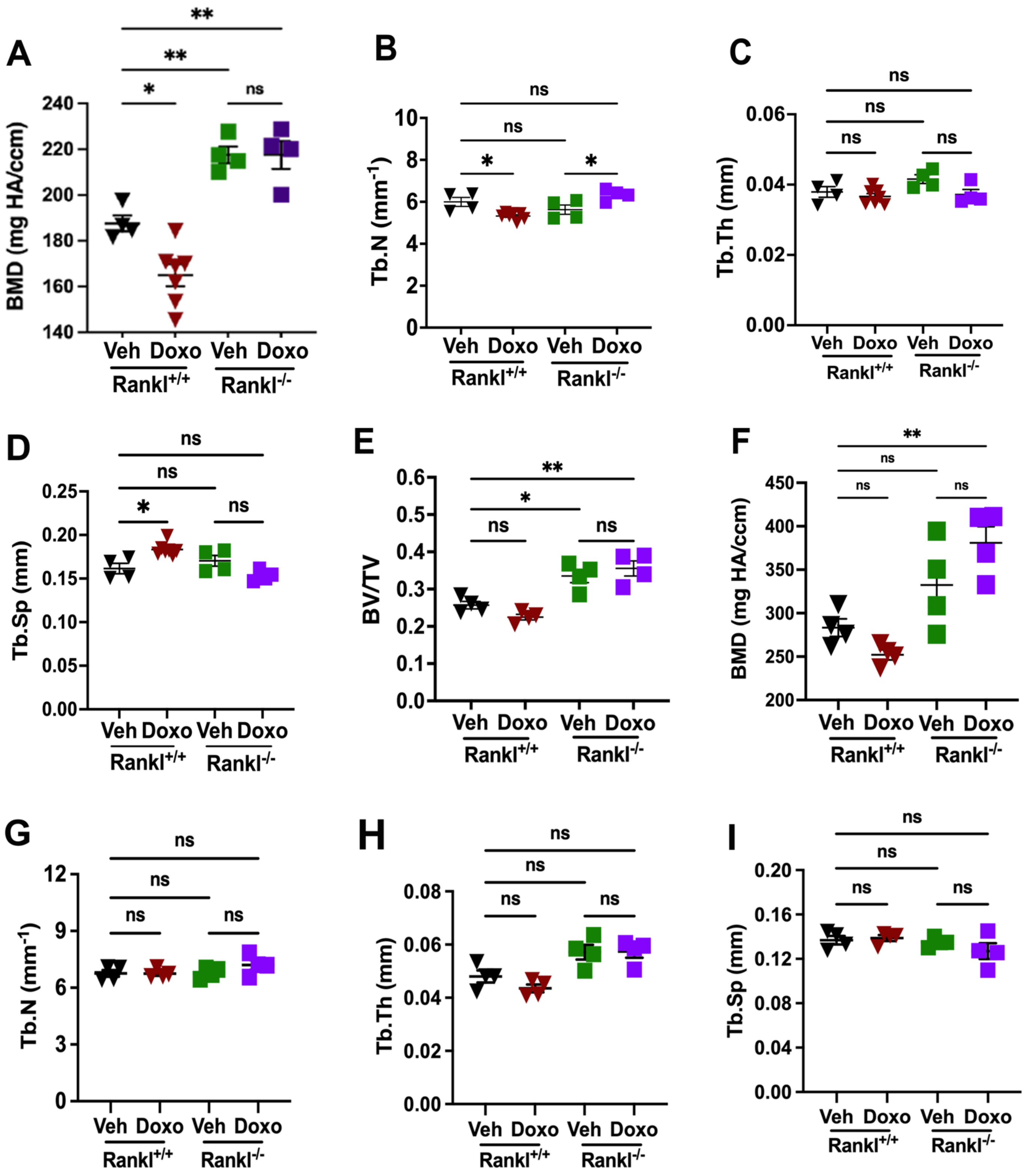
A
BMD (mg HA/ccm)
240
220
200
180
160
140
**
**
*
ns
Veh Doxo Veh Doxo
Rankl+/+ Rankl-/-
B
Tb.N (mm-1)
10
8
6
4
2
0
ns
ns
*
*
Veh Doxo Veh Doxo
Rankl+/+ Rankl-/-
C
Tb.Th (mm)
0.06
0.04
0.02
0.00
ns
ns
ns
ns
Veh Doxo Veh Doxo
Rankl+/+ Rankl-/-
D
Tb.Sp (mm)
0.25
0.20
0.15
0.10
0.05
0.00
ns
ns
*
ns
Veh Doxo Veh Doxo
Rankl+/+ Rankl-/-
E
BV/TV
0.6
0.5
0.4
0.3
0.2
0.1
0.0
**
*
ns
ns
Veh Doxo Veh Doxo
Rankl+/+ Rankl-/-
F
BMD (mg HA/ccm)
450
400
350
300
250
200
**
ns
ns
ns
Veh Doxo Veh Doxo
Rankl+/+ Rankl-/-
G
Tb.N (mm-1)
12
9
6
3
0
ns
ns
ns
ns
Veh Doxo Veh Doxo
Rankl+/+ Rankl-/-
H
Tb.Th (mm)
0.08
0.06
0.04
0.02
0.00
ns
ns
ns
ns
Veh Doxo Veh Doxo
Rankl+/+ Rankl-/-
I
Tb.Sp (mm)
0.20
0.16
0.12
0.08
0.04
0.00
ns
ns
ns
ns
Veh Doxo Veh Doxo
Rankl+/+ Rankl-/-

Fig. S11. RANKL deletion in adipo-lineage cells prevent chemotherapy-induced bone loss.

(A-D) µCT analysis shows bone mineral density (BMD), trabecular number (Tb. N), trabecular thickness (Tb. Th), and trabecular separation (Tb.Sp) in 12-week-old female adipo-Cre-/Rankl$^{fl/fl}$ (Rankl$^{+/+}$) and ADQ-Cre+/Rankl$^{fl/fl}$ (Rankl$^{-/-}$) mice. n = 4 mice (Rankl$^{+/+}$, Veh), n = 7 mice (Rankl$^{+/+}$, Doxo), n = 4 mice (Rankl$^{-/-}$, Veh) and n = 4 mice (Rankl$^{-/-}$, Doxo).

(E-I) µCT analysis shows trabecular bone volume to total volume (BV/TV) with other bone parameters in 12-week-old male adipo-Cre-/Rankl$^{fl/fl}$ (Rankl$^{+/+}$) and adipo-Cre+/Rankl$^{fl/fl}$ (Rankl$^{-/-}$) mice. n = 4 mice/group.

Data are represented as mean ± SEM. *P < 0.05; **P < 0.01; ns = not significant as determined by one-way ANOVA with Tukey.

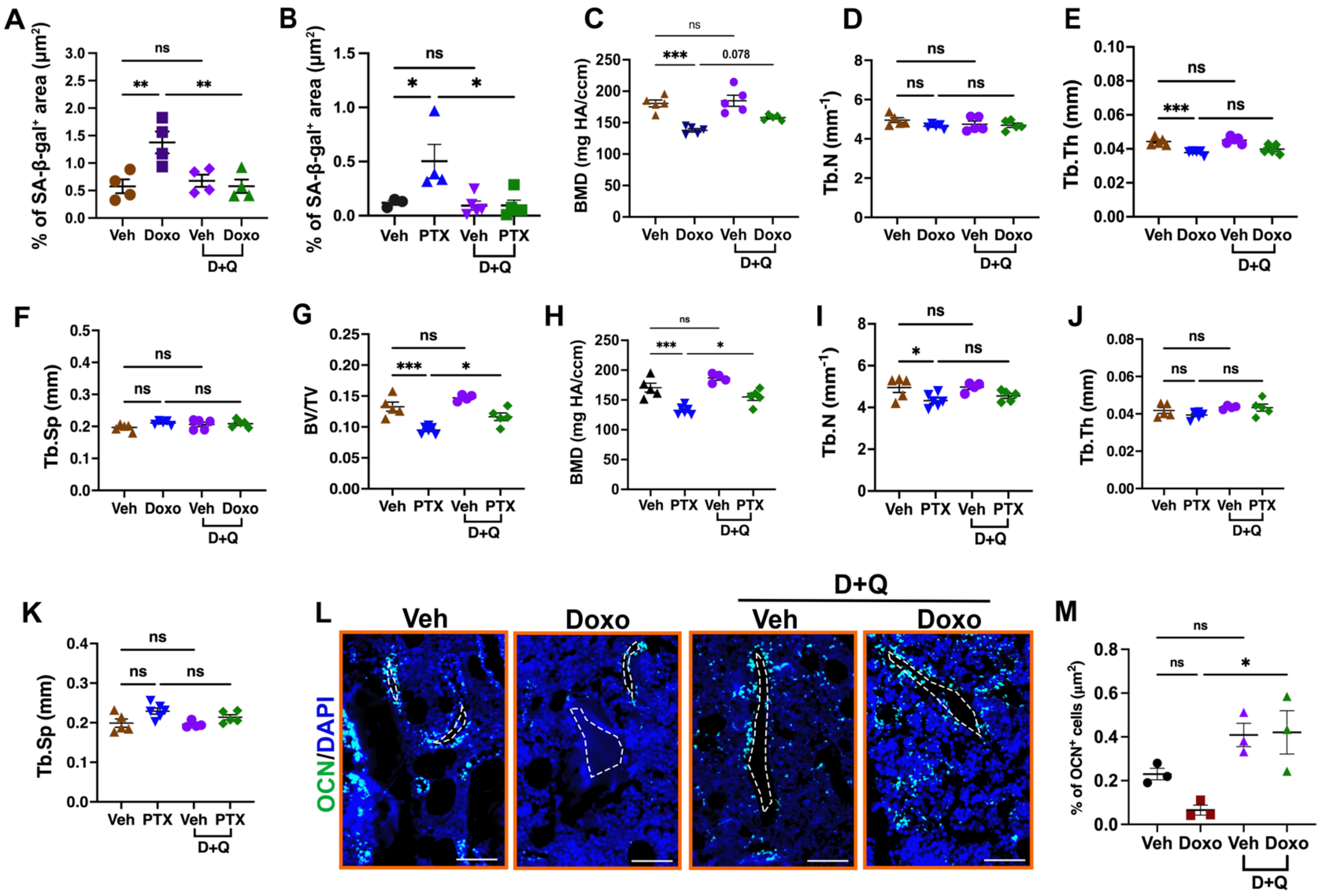
A
% of SA-β-gal⁺ area (μm²)
Veh Doxo Veh Doxo
D+Q
B
% of SA-β-gal⁺ area (μm²)
Veh PTX Veh PTX
D+Q
C
BMD (mg HA/ccm)
0.078
Veh Doxo Veh Doxo
D+Q
D
Tb.N (mm⁻¹)
E
Tb.Th (mm)
F
Tb.Sp (mm)
G
BV/TV
H
BMD (mg HA/ccm)
I
Tb.N (mm⁻¹)
J
Tb.Th (mm)
K
Tb.Sp (mm)
L
Veh
Doxo
D+Q
OCN/DAPI
M
% of OCN⁺ cells (μm²)
ns

Fig. S12. Senolytics prevent chemotherapy-induced bone loss.

(A and B) Quantification of SA-β-gal positive area in Doxo- and PTX-treated mice. n = 4/group (**A**). n = 3 mice (Veh), 4 (PTX), 5 mice (Veh+D/Q), and 4 mice (PTX+D/Q) (**B**).

(C-F) Representative μCT quantitative analyses of bone mineral density (BMD), trabecular number (Tb.N), trabecular thickness (Tb. Th) and trabecular spacing (Tb. Sp). n = 5 mice/group.

(G-K) Representative μCT quantitative analyses of trabecular bone volume to total bone volume (BV/TV), bone mineral density (BMD), trabecular number (Tb.N), trabecular thickness (Tb. Th), trabecular spacing (Tb. Sp) in PTX-treated mice. n = 5 mice/group.

(L and M) Immunofluorescence staining of osteocalcin (OCN) and quantification of the OCN+ osteoblast area. Dotted line indicates trabecular bone. Scale bar: 50μm. n = 3 mice/group.

Data are represented as mean ± SEM. *$P < 0.05$; **$P < 0.01$; ***$P < 0.001$; ns = not significant as determined by one-way ANOVA with Tukey test.

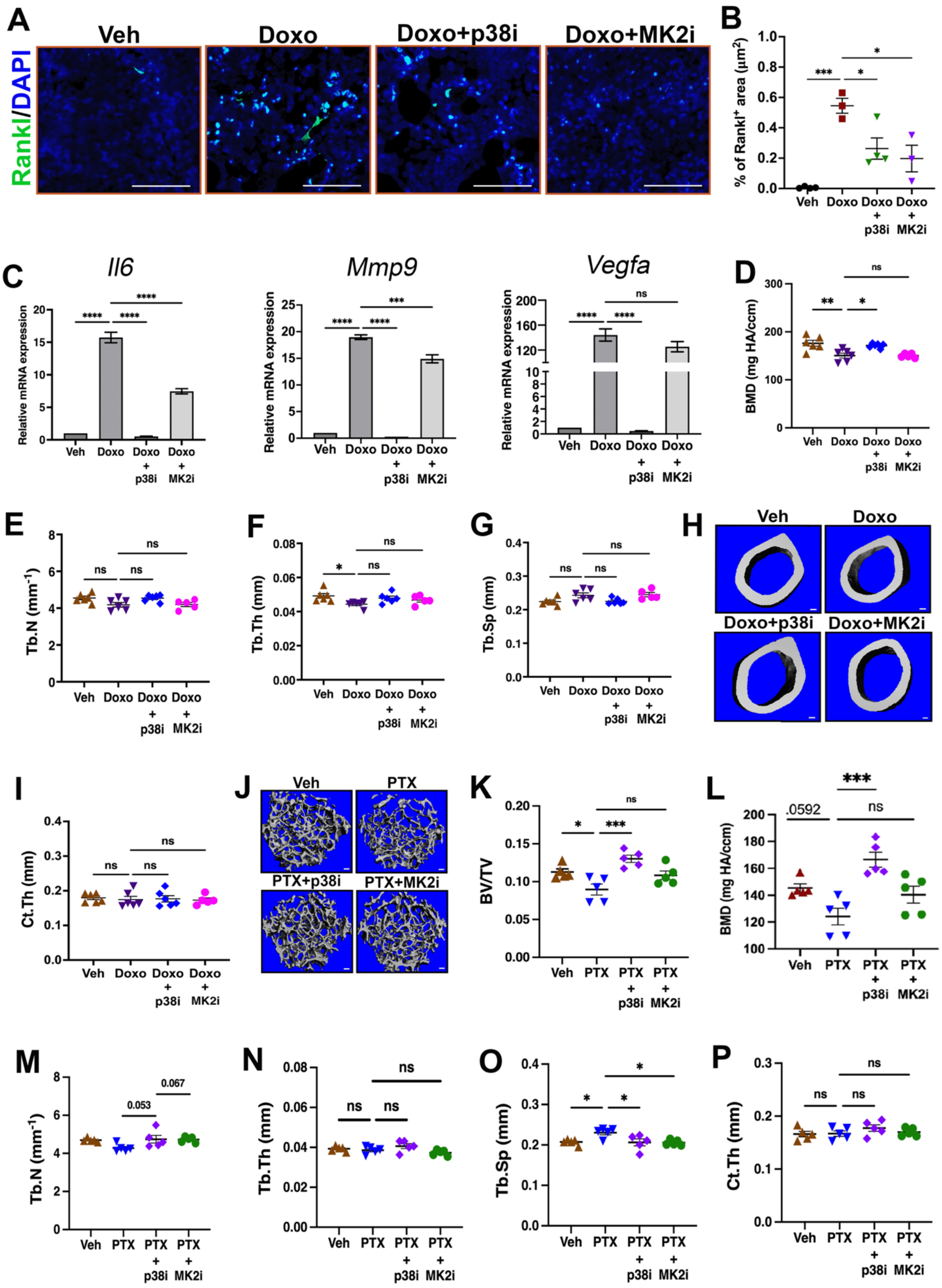
A
Veh
Doxo
Doxo+p38i
Doxo+MK2i
Rankl/DAPI
B
% of Rankl+ area (μm2)
***
*
*
Veh
Doxo
Doxo + p38i
Doxo + MK2i
C
Il6
Mmp9
Vegfa
Relative mRNA expression
****
***
ns
D
BMD (mg HA/ccm)
**
*
ns
E
Tb.N (mm-1)
F
Tb.Th (mm)
G
Tb.Sp (mm)
H
Veh
Doxo
Doxo+p38i
Doxo+MK2i
I
Ct.Th (mm)
J
Veh
PTX
PTX+p38i
PTX+MK2i
K
BV/TV
L
BMD (mg HA/ccm)
.0592
M
Tb.N (mm-1)
0.053
0.067
N
Tb.Th (mm)
O
Tb.Sp (mm)
P
Ct.Th (mm)
PTX
PTX + p38i
PTX + MK2i

Fig. S13. Senomorphics prevent chemotherapy-induced bone loss.

(A and B) Immunofluorescence staining showing RANKL (green) positive cells in femoral bone sections of 12 weeks-old wild type C57BL/6 mice. DAPI stained nuclei are blue. Quantification of the percentage of RANKL positive area. Scale bar: 50μm. n = 4 mice (Veh), 3 mice (Doxo), 4 mice (Doxo+p38i), and 3 mice (Doxo+MK2i).

(C) mRNA expression of SASP factors (*Il6, MMP9 and Vegfa*) in bone-resident fraction under indicated conditions, determined by RT-qPCR. Actin, cyclophilin, TBP and tubulin were used as housekeeping genes. n = 3 mice were combined/group.

(D-G) μCT quantitative analyses of bone mineral density (BMD), trabecular number (Tb.N), trabecular thickness (Tb. Th) and trabecular spacing (Tb. Sp). n = 6 mice (Veh), 6 mice (Doxo), 6 mice (Doxo+p38i), and 5 mice (Doxo+MK2i).

(H and I) Representative μCT images and quantitative analyses of cortical bone thickness (Ct. Th). Scale bar=100μm. n = 6 mice (Veh), 6 mice (Doxo), 6 mice (Doxo+p38i), and 5 mice (Doxo+MK2i).

(J-P) Representative μCT images and quantitative analyses of trabecular bone volume to total bone volume (BV/TV), bone mineral density (BMD), trabecular number (Tb.N), trabecular thickness (Tb. Th), trabecular spacing (Tb. Sp) and cortical bone thickness (Ct. Th) in PTX-treated mice. n = 5 mice/group.

Data are represented as mean ± SEM. *$P < 0.05$; **$P < 0.01$; ***$P < 0.001$; ****$P < 0.0001$; ns = not significant as determined by one-way ANOVA with Tukey test.

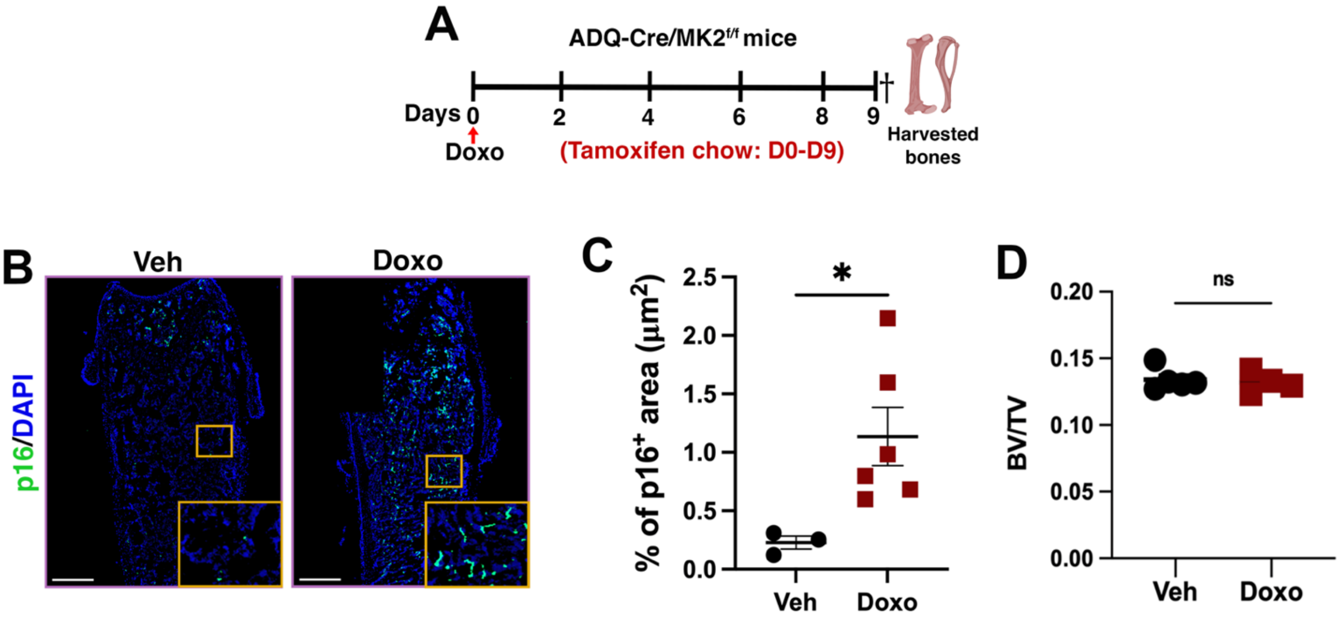
A
ADQ-Cre/MK2f/f mice
Days 0 2 4 6 8 9
Doxo
(Tamoxifen chow: D0-D9)
Harvested bones
B
Veh
Doxo
p16/DAPI
C
% of p16+ area (μm2)
2.5
2.0
1.5
1.0
0.5
0.0
*
Veh
Doxo
D
BV/TV
0.20
0.15
0.10
0.05
0.00
ns
Veh
Doxo

Fig. S14. ADQ-Cre^{ERT2} mediated MK2 deletion prevents chemotherapy-induced bone loss

(A) The schematic illustrates the time points for tamoxifen chow and other treatments in ADQ-Cre/MK2$^{f/f}$ mice. Schematic was created in BioRender. Stewart, S. (2025) https://BioRender.com/71wz45j.

(B and C) Immunofluorescence staining for p16 in femur sections obtained from ADQ-Cre+/MK2$^{fl/fl}$ mice. p16+ cells are visualized in green, and nuclei are counterstained with DAPI (blue). Scale bar: 50μm. n = 3 mice (Veh) and 6 mice (Doxo).

(D) μCT quantitative analyses of trabecular bone volume to total bone volume (BV/TV), trabecular number (Tb.N) in ADQ-Cre+/MK2$^{f/f}$ mice. n = 5 mice/group.

Data are represented as mean ± SEM. *$P < 0.05$; ns = not significant as determined by unpaired two-tailed Student's *t*-tests.

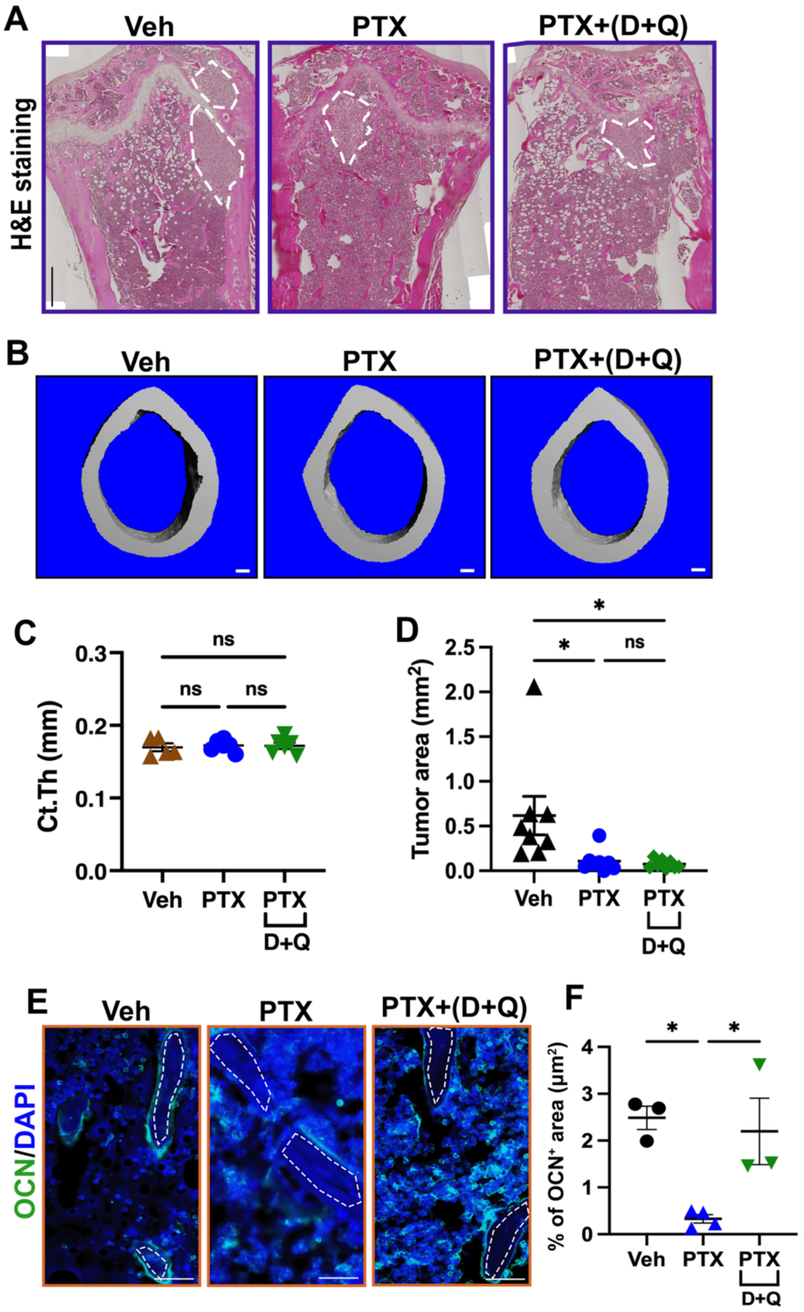

A
Veh
PTX
PTX+(D+Q)
H&E staining
B
Veh
PTX
PTX+(D+Q)
C
Ct.Th (mm)
0.0
0.1
0.2
0.3
ns
ns
ns
Veh
PTX
PTX
D+Q
D
Tumor area (mm2)
0.0
0.5
1.0
1.5
2.0
2.5
*
*
ns
Veh
PTX
PTX
D+Q
E
Veh
PTX
PTX+(D+Q)
OCN/DAPI
F
% of OCN+ area (μm2)
0
1
2
3
4
*
*
Veh
PTX
PTX
D+Q

Fig. S15. Senolytics prevent chemotherapy-induced bone loss in tumor bearing mice.

(A) H&E staining illustrates tumor burden in bone (demarcated by white hashed lines) under the indicated treatment condition. Scale bar: 500μm.

(B and C) μCT analysis shows cortical thickness (Ct. Th) of femurs. n = 5 mice (Veh), 6 mice (PTX), and 6 mice (PTX+D/Q).

(D) Quantitative assessment of tumor area in the TRAP-stained femurs. n = 8 mice/group.

(E and F) Immunofluorescence staining of osteocalcin and quantification of the number of OCN+ osteoblast area. Dotted line indicates trabecular bone. Scale bar: 50μm. n = 3 mice (Veh), 4 mice (PTX), and 3 mice (PTX+D/Q).

Data are represented as mean ± SEM. **P* < 0.05; ns = not significant as determined by one-way ANOVA with Tukey test.

Supplementary Table 1

Reagent or resource	Source	Identifier
**Antibodies**		
Rabbit anti-p16	Invitrogen	Cat# PA1-46220; RRID: AB_2291616
Rabbit anti-EBF3	Abcam	Cat# AB207705; UniProt: Q9H4W6
Rabbit anti-OCN	Proteintech	Cat# 23418-1AP; RRID: AB_2879275
Rabbit anti-RANKL	Bioss	Cat# BS-0747R; RRID: AB_10855901
Mouse anti- PPARg	Proteintech	Cat# 669361; RRID: AB_2882260
Rabbit anti-S100a4	Millipore Sigma	Cat# SAB5700127; UniProt: P26447
Rabbit anti-Sox9	Abcam	Cat# ab186966; RRID AB_2728660
Mouse anti-CD45-APC	BioLegend	Cat# 103112; RRID: AB_312976
Mouse anti-CD71-APC	BioLegend	Cat# 113819; RRID: AB_2728134
Mouse anti-Ter119-APC	BioLegend	Cat# 116211; RRID: AB_313712
Rat anti-CD31-APC	Invitrogen	Cat# 170311-80; RRID: AB_657736
Mouse anti-Pdgfrb-PE	BioLegend	Cat# 136005; RRID: AB_1953271
Rat anti-Sca1-APC	BioLegend	Cat# 108112 D7; RRID: AB_313348

Mouse anti-CD45.1-eFluor 450	Thermo Scientific	Cat# 48-0453-80; RRID: AB_1272225
Mouse anti-CD45.2-APC	Thermo Scientific	Cat# 17-0454-81; RRID: AB_469399
Mouse-CD45 microbeads	Miltenyi Biotec	Cat# 130-052-301; RRID: AB_2877061
Fc blocker	BD Pharmingen	Cat# 553142; RRID: AB_394656
Secondary goat anti–rabbit Alexa Fluor	Invitrogen	Cat# A-11011; RRID: AB_143157
Secondary goat anti–mouse Alexa Fluor	Invitrogen	Cat# A-11001; RRID: AB_2534069
**Chemicals, peptides, and recombinant proteins**		
AP20187	APExBIO	Cat# B1274
Doxorubicin	MedChemExpress	Cat# HY-15142/CS-1239
Paclitaxel	TOCRIS	Cat# 1097
Dasatinib	LC Laboratory	Cat# D-3307
Quercetin	Sigma	Cat# Q4951
Diphtheria Toxin	Biological List Laboratories	Cat# 150
SPiDER Probe	Dojindo Laboratory	Cat# SG02
BODIPY	Invitrogen	Cat# D3922
Calcein	Sigma	Cat# C0875
Alizarin red	Sigma	Cat# A3882
Oil red O dye	Sigma	Cat# 00625
Naphthol AS-MX phosphate	Sigma	Cat# N4875
Fast Red TR Salt hemi salt	Sigma	Cat# 368881
X-Gal	Sigma	Cat# B4252
SlowFade Gold antifade reagent with DAPI	Invitrogen	Cat# S36939

BCIP/NBT	SigmaFast	Cat# B5655
10% NBF	Epredia	Ref 5705
DMEM F12	Gibco	Cat# 11320033
b-glycerophosphate	APExBIO	Cat# C4347
Indomethacin	Sigma	Cat# I8280
Dexamethasone	Sigma	Cat# D4902
3-Isobutyl-1-methylxanthine	Sigma	Cat# I5879
Insulin	Sigma	Cat# I9278
Trypsin-EDTA (0.25%)	Gibco	Cat# 25200072
1x Pen Strep	Gibco	Cat# 15140122
PBS	Sigma	Cat# D1408
DMEM medium	Sigma	Cat# D6429
FBS (Fetal Bovine Serum)	Sigma	Cat# F1051
EDTA	Sigma	Cat# E5134
TRIzol Reagent	Invitrogen	Cat# 15596-026
BSA	Sigma	Cat# D9663
Tamoxifen chow	Inotiv	Cat# TD.130858
Collagenase	Sigma	Cat# C0130
Molecular grade water	Corning	Cat# 46-000-CM
Poly (ethylene glycol)	Sigma	Cat# 91893
PHOSAL 50 PG	Lipoid	Batch: 368315-3180028/041
1X TE solution	Integrated DNA Technologies	Cat# 11-05-01-09
O.C.T. compound	Fisher Health Care	Cat# 4585
**Critical commercial assays**		
RiboPure Kit	Invitrogen	Cat# AM1924
RNeasy Mini kit	Qiagen	Cat# 74004
Taqman PrimeTime Gene Expression Master Mix Kit	Integrated DNA Technologies	Cat# 1055771

M.O.M. Kit	Vector Laboratories	Cat# PK-2200
Navy RINO Lysis Kit	Next Advance	Lot: Cap8320
scRNA Seq kit	10x Genomics	
**Deposited data**		
scRNA-Seq data	GEO	GEO: GSE289491
scRNA-Seq data	GEO	GEO: GSE230295 [18]
**Experimental models: Organisms/strains**		
INK-ATTAC	Jan van Deursen	Mouse provided by Unity Biotechnology
p16Ink4a-CreERT2neo mice (p16-Cre^{ERT2})	Makoto Nakanishi	Omori et al., 2020[17]
ROSA26-CAG-lsl-tdTomato mice (tdTomato)	The Jackson Laboratory	Stock No: 007905
Tg(Col1a1*2.3-GFP)1Rowe/J (Col1a1*2.3-GFP)	The Jackson Laboratory	Stock No: 013134
Tg(Adipoq-cre)1Evdr/J (ADQ-Cre)	The Jackson Laboratory	Stock No: 010803
Tg(Adipoq-icre/ERT2)1Soff/J (ADQ-Cre^{ERT2})	The Jackson Laboratory	Stock No: 025124
INK-QR (Lox-Stop-Lox)	Developed by the Stewart and DeNardo laboratories	Mouse provided by S. Stewart's group
$Tnfsf11^{tm1c/d}$ (Lox-Stop-Lox)	Marco Colonna laboratories	Mouse provided by S. Teitelbaum's group
$Mapkapk2^{tm2.1Yaff}$/J (Lox-Stop-Lox)	The Jackson Laboratory	Stock No: 032450
Cxcl12-GFP reporter mice	Takashi Nagasawa	Toshiaki Ara et al., 2003
Gt(ROSA)$26Sor^{tm1(HBEGF)Awai/J}$ (Lox-Stop-Lox-ROSA DTR)	The Jackson Laboratory	Stock No: 007900
C57BL/6J	The Jackson Laboratory	Stock No: 000664
B6(Cg)-Tyr^{c-2J}/J (Albino mice)	The Jackson Laboratory	Stock No: 000058

Oligonucleotides		
RT-qPCR Cdkn2a Forward-Primer: 5’-AACTCTTTCGGTCGTACCCC-3’; Reverse-Primer: 5’-TCCTCGCAGTTCGAATCTG-3’.	Integrated DNA Technologies	Ref. No.:260287447/260287448
RT-qPCR p21 Forward-Primer: 5’-GAAGAGACAACGGCACACT-3’; Reverse-Primer: 5’-CAGATCCACAGCGATATCCAG-3’.	Integrated DNA Technologies	Ref. No.:260287445/260287446
RT-qPCR *Il6* Forward-Primer: 5’-AGCCAGAGTCCTTCAGAGA-3’; Reverse-Primer: 5’-TCCTTAGCCACTCCTTCTGT-3’.	Integrated DNA Technologies	Ref. No.: 205234847/205234848
RT-qPCR *Rankl* Forward-Primer: 5’-TCCCGCTCCATGTTCCT-3’; Reverse-Primer: 5’-AGTGCTGTCTTCTGATATTCTGT-3’.	Integrated DNA Technologies	Ref. No.:410311816
RT-qPCR Opg Forward-Primer: 5’-TGGTATAATCTTGGTAGGAACAGC-3’; Reverse-Primer: 5’-ATGCAACACATGACAACGTG-3’	Integrated DNA Technologies	Ref. No.: 210949421
RT-qPCR Dmp1 Forward-Primer: 5’- GTCACTATTTGCCTGTCCCT-3’; Reverse-Primer: 5’-TCCCAGTTGCCAGATACCA-3’	Integrated DNA Technologies	Ref. No.: 210949409

RT-qPCR Col1a1 Forward-Primer: 5'- CATTGTGTAT GCAGCTGACTTC-3'; Reverse-Primer: 5'-CGCAA AGAGTCTACATGTCTAGG-3'.	Integrated DNA Technologies	Ref. No.: 258778385
RT-qPCR PPARg Forward-Primer: 5'-TGCAGGTTCTA CTTTGATCGC-3'; Reverse-Primer: 5'-CTGCTCCACAC TATGAAGACAT-3'.	Integrated DNA Technologies	Ref. No.:205086994
RT-qPCR Mmp9 Forward-Primer: 5'-GTGGGAGGTAT AGTGGGACA-3'; Reverse-Primer: 5'-GACATAGACGG CATCCAGTATC-3'.	Integrated DNA Technologies	Ref. No.: 210947632
RT-qPCR Vegfa Forward-Primer: 5'-GACTTCTGCTCT CCTTCTGTC-3'; Reverse-Primer: 5'- CCGAAACCATG AACTTTCTGC-3'.	Integrated DNA Technologies	Ref. No.: 210947616
RT-qPCR Actin Forward Primer: 5'-TTTCCAGCCTTC CTTCTTGG-3'; Reverse-Primer: 5'-GGCATAGAGGT CTTTACGGATG-3'.	Integrated DNA Technologies	Ref. No.: 299974586/299974587
RT-qPCR Tubulin Forward Primer: 5'-TCTTGTCACTTG GCATCTGG-3'; Reverse-Primer: 5'-CGCGAAGCAGC AACCAT-3'.	Integrated DNA Technologies	Ref. No.: 410593635/2736866247

RT-qPCR Cyclophilin Forward-Primer: 5'-TTCAC CTTCCCAAAGACCAC-3'; Reverse-Primer: 5'-CAAAC ACAAACGGTTCCCAG-3'.	Integrated DNA Technologies	Ref. No.: 462233404/462233405
RT-qPCR TBP Forward-Primer: 5'-CCCTTCACCA ATGACTCCTATG-3'; Reverse-Primer: 5'-CAGCC AAGATTCACGGTAGAT-3'.	Integrated DNA Technologies	Ref. No.: 320232457/320232458
**Software and algorithms**		
Bioquant Osteo v7 10.10	Bioquant	RRID: SCR_016423
GraphPad Prism 10	GraphPad	RRID: SCR_002798
RStudio/Seurat package	Posit PBC	51, 55
HALO (v3.3.2541.231)	Indica Lab	N/A
