# Supplementary material for: Chemotherapy-induced adipo-lineage cell senescence drives bone loss
Source: Nat Commun. 2025 Dec 30;17:1042. doi: 10.1038/s41467-025-67793-3 (PMC12848019; doi:10.1038/s41467-025-67793-3)
Supplement: Supplementary file 2 — Reporting Summary [file 41467_2025_67793_MOESM2_ESM.pdf]

nature portfolio

Corresponding author(s): Sheila A Stewart

Last updated by author(s): Oct 13, 2025

Reporting Summary

Nature Portfolio wishes to improve the reproducibility of the work that we publish. This form provides structure for consistency and transparency in reporting. For further information on Nature Portfolio policies, see our Editorial Policies and the Editorial Policy Checklist.

Please do not complete any field with "not applicable" or n/a. Refer to the help text for what text to use if an item is not relevant to your study.
For final submission: please carefully check your responses for accuracy; you will not be able to make changes later.

Statistics

For all statistical analyses, confirm that the following items are present in the figure legend, table legend, main text, or Methods section.

n/a	Confirmed	
☐	☒	The exact sample size (n) for each experimental group/condition, given as a discrete number and unit of measurement
☐	☒	A statement on whether measurements were taken from distinct samples or whether the same sample was measured repeatedly
☐	☒	The statistical test(s) used AND whether they are one- or two-sided<br>*Only common tests should be described solely by name; describe more complex techniques in the Methods section.*
☐	☒	A description of all covariates tested
☐	☒	A description of any assumptions or corrections, such as tests of normality and adjustment for multiple comparisons
☐	☒	A full description of the statistical parameters including central tendency (e.g. means) or other basic estimates (e.g. regression coefficient) AND variation (e.g. standard deviation) or associated estimates of uncertainty (e.g. confidence intervals)
☐	☒	For null hypothesis testing, the test statistic (e.g. F, t, r) with confidence intervals, effect sizes, degrees of freedom and P value noted<br>*Give P values as exact values whenever suitable.*
☒	☐	For Bayesian analysis, information on the choice of priors and Markov chain Monte Carlo settings
☒	☐	For hierarchical and complex designs, identification of the appropriate level for tests and full reporting of outcomes
☒	☐	Estimates of effect sizes (e.g. Cohen's d, Pearson's r), indicating how they were calculated

Our web collection on statistics for biologists contains articles on many of the points above.

Software and code

Policy information about availability of computer code

Data collection	A publicly available human bone scRNA-seq dataset (GEO: GSE230295) from acute lymphoblastic leukemia patients were used. Details are mentioned in the manuscript.
Data analysis	RStudio/Seurat package (Posit PBC)

For manuscripts utilizing custom algorithms or software that are central to the research but not yet described in published literature, software must be made available to editors and reviewers. We strongly encourage code deposition in a community repository (e.g. GitHub). See the Nature Portfolio guidelines for submitting code & software for further information.

Data

Policy information about availability of data

All manuscripts must include a data availability statement. This statement should provide the following information, where applicable:

- Accession codes, unique identifiers, or web links for publicly available datasets
- A description of any restrictions on data availability
- For clinical datasets or third party data, please ensure that the statement adheres to our policy

Data Availability
Source data are provided with this paper. The scRNA-seq datasets analyzed in this study are available through the NCBI Gene Expression Omnibus (GEO) under the accession codes GSE289491 (samples derived from mice) and GSE230295 (samples derived from acute lymphoblastic leukemia patients) 18. Raw data including

micrographs can be found on Zendo, 10.5281/zenodo.17314594.

Research involving human participants, their data, or biological material

Policy information about studies with human participants or human data. See also policy information about sex, gender (identity/presentation), and sexual orientation and race, ethnicity and racism.

Reporting on sex and gender	A publicly available human bone scRNA-seq dataset (GEO: GSE230295) from male acute lymphoblastic leukemia patients was analyzed, as described in the manuscript.
Reporting on race, ethnicity, or other socially relevant groupings	n/a
Population characteristics	n/a
Recruitment	n/a
Ethics oversight	n/a

Note that full information on the approval of the study protocol must also be provided in the manuscript.

Field-specific reporting

Please select the one below that is the best fit for your research. If you are not sure, read the appropriate sections before making your selection.

☒ Life sciences ☐ Behavioural & social sciences ☐ Ecological, evolutionary & environmental sciences

For a reference copy of the document with all sections, see nature.com/documents/nr-reporting-summary-flat.pdf

Life sciences study design

All studies must disclose on these points even when the disclosure is negative.

Sample size	In several experiments, 3–5 mice per group were required to achieve 90% power at a 5% significance level to test the null hypothesis of no difference.
Data exclusions	n/a
Replication	Analyses were performed by another independent researcher who was blind to sample identity. All replications were successful.
Randomization	Mice were randomly assigned to the control groups and treated groups.
Blinding	Analyzed by a secondary researcher who was blind for the sample identity.

Reporting for specific materials, systems and methods

We require information from authors about some types of materials, experimental systems and methods used in many studies. Here, indicate whether each material, system or method listed is relevant to your study. If you are not sure if a list item applies to your research, read the appropriate section before selecting a response.

Materials & experimental systems

n/a	Involved in the study	
☐	☒	Antibodies
☐	☒	Eukaryotic cell lines
☒	☐	Palaeontology and archaeology
☐	☒	Animals and other organisms
☐	☒	Clinical data
☒	☐	Dual use research of concern
☒	☐	Plants

Methods

n/a	Involved in the study	
☒	☐	ChIP-seq
☐	☒	Flow cytometry
☒	☐	MRI-based neuroimaging

Antibodies

Antibodies used	p16, RANKL, OCN, PPARG, EBF3, SOX9, S100a4, CD71, CD45, Ter119, CD31, Pdgfrb, Sca1, Fc-blocker. All details are mentioned in the manuscript table.
Validation	Source and identifier details for antibodies are mentioned in the manuscript table.

Eukaryotic cell lines

Policy information about cell lines and Sex and Gender in Research

Cell line source(s)	Bo-1 PyMT derived from mouse. Gifted by Dr. Kathy Weilbaecher's group.
Authentication	STR
Mycoplasma contamination	Mycoplasma contamination was tested and results showed cells are contamination free.
Commonly misidentified lines (See ICLAC register)	n/a

Animals and other research organisms

Policy information about studies involving animals; ARRIVE guidelines recommended for reporting animal research, and Sex and Gender in Research

Laboratory animals	Mice (Mus musculus) were used in this study
Wild animals	n/a
Reporting on sex	This study included both female and male mice, with details provided in the manuscript where relevant.
Field-collected samples	n/a
Ethics oversight	All animal experiments were performed in compliance with Washington University in St. Louis's Animals Studies Committee. All animal procedures were approved by Washington, University's Institutional Animal Care and Use Committees (IACUC). The maximal tumor size permitted was 2 cm in diameter. Experimental endpoints were determined by tumor size (not exceeding 2 cm) or by clinical signs associated with metastatic burden, including weight loss exceeding 20% of body weight

Note that full information on the approval of the study protocol must also be provided in the manuscript.

Clinical data

Policy information about clinical studies

All manuscripts should comply with the ICMJE guidelines for publication of clinical research and a completed CONSORT checklist must be included with all submissions.

Clinical trial registration	n/a
Study protocol	n/a
Data collection	n/a
Outcomes	n/a

Plants

Seed stocks	n/a
Novel plant genotypes	n/a
Authentication	n/a

Flow Cytometry

Plots

Confirm that:

☒ The axis labels state the marker and fluorochrome used (e.g. CD4-FITC).

☒ The axis scales are clearly visible. Include numbers along axes only for bottom left plot of group (a 'group' is an analysis of identical markers).

☒ All plots are contour plots with outliers or pseudocolor plots.

☒ A numerical value for number of cells or percentage (with statistics) is provided.

Methodology

Sample preparation	Bones were gently ground and then cut into small fragments. Fragments were transferred and digested with fresh 2 mg/ml collagenase (Sigma, C0130) in DMEM/F12 on a rotating water bath at 37°C for 30 min. Following the first digestion, the released cell suspension was filtered through a 70-μm nylon mesh into a collection tube and placed on ice. A second digestion of the remaining fragments was performed and the cell suspension was filtered into the same collection tube. Reactions were quenched with FACS buffer (PBS plus 0.5% BSA) with 2mM EDTA. Following FACS buffer wash and RBC lysis, dissociated cells were labeled with anti-CD45 magnet microbeads (Miltenyi Biotech) and enriched into a CD45+ fraction and CD45- fraction by MACS (magnetic-activated cell sorting, Miltenyi Biotech). Cells were stained with fluorochrome antibodies for 20 min on ice for cell sorting. Sorted cells were either subject to 10X for single cell RNA-seq or RT-qPCR where total RNA was extracted using the RiboPure™ RNA Purification Kit (AM1924, Invitrogen). Sorting purity/enrischment was confirmed by qRT-PCR of marker genes.
Instrument	MoFlo cell sorter and BD FACS Aria III Cell Sorter
Software	FlowJo.
Cell population abundance	After cell sorting, a sufficient number of cells were collected and subjected to RNA isolation under RNase-free conditions.
Gating strategy	Cells were gated to exclude debris, doublets, and dead cells, followed by identification of specific populations based on fluorescence marker expression. Details are added in manuscript and raw data is deposited in source file

☒ Tick this box to confirm that a figure exemplifying the gating strategy is provided in the Supplementary Information.

This checklist template is licensed under a Creative Commons Attribution 4.0 International License, which permits use, sharing, adaptation, distribution and reproduction in any medium or format, as long as you give appropriate credit to the original author(s) and the source, provide a link to the Creative Commons license, and indicate if changes were made. The images or other third party material in this article are included in the article's Creative Commons license, unless indicated otherwise in a credit line to the material. If material is not included in the article's Creative Commons license and your intended use is not permitted by statutory regulation or exceeds the permitted use, you will need to obtain permission directly from the copyright holder. To view a copy of this license, visit http://creativecommons.org/licenses/by/4.0/
